# New Mild and Simple Approach to Isothiocyanates: A Class of Potent Anticancer Agents

**DOI:** 10.3390/molecules22060773

**Published:** 2017-06-01

**Authors:** Bingling Luo, Jiankang Wang, Xiaobing Li, Wenhua Lu, Jing Yang, Yumin Hu, Peng Huang, Shijun Wen

**Affiliations:** 1 Sun Yat-Sen University Cancer Center; State Key Laboratory of Oncology in South China; Collaborative Innovation Center for Cancer Medicine; Sun Yat-Sen University, 651 Dongfeng East Road, Guangzhou 510060, China; luobl@sysucc.org.cn (B.L.); wangjk6@mail2.sysu.edu.cn (J.W.); lixiaobing0629@126.com (X.L.); luwenh@sysucc.org.cn (W.L.); yangjing@sysucc.org.cn (J.Y.); huym@sysucc.org.cn (Y.H.); huangpeng@sysucc.org.cn (P.H.); 2 School of Pharmaceutical Sciences, Sun Yat-sen University, 132 Waihuan East Road, Guangzhou 510006, China

**Keywords:** isothiocyanate, anticancer, reactive oxygen species, glutathione

## Abstract

In our current work, acetyl chloride-mediated synthesis of phenethyl isothiocyanate (PEITC) derivatives proves to be convenient and provides the expected products at good to excellent yields. Biological evaluation and structure-activity relationship analysis found that the novel compound **7** showed the best anticancer activity against human cancer cell line Panc1 and HGC27 compared with PEITC. Compounds **6** and **7** induced more apoptosis in pancreatic cancer cells but less toxicity in non-cancer cells. Further biological study demonstrated that **7** substantially increased intracellular reactive oxygen species (ROS) and depleted glutathione (GSH), leading to an oxidative stress to kill cancer cell.

## 1. Introduction

Isothiocyanates are important natural compounds found in cruciferous vegetables such as watercress and broccoli, and they have shown cancer prevention effects [1,2]. Meanwhile, it is also reported that these natural isothiocyanates can induce apoptosis of cancer cells, although its mechanism is still under debate [3,4,5,6]. Among these reported natural compounds, one of the most studied is phenethyl isothiocyanate (PEITC). Packham’s group found that PEITC could target mTORC1 activity in leukemia cells [7,8], and Chung and his co-workers reported that isothiocyanates selectively depleted mutant p53, compared with wild type p53 [9]. In our previous study, PEITC showed a great selectivity in killing oncogenically transformed cells over normal cells through a reactive oxygen species (ROS)-mediated mechanism [10,11,12], indicating that PEITC could hold therapeutic promise for the treatment of cancer patients. Indeed, PEITC has been registered for clinical trials for cancer prevention and treatment (ClinicalTrials.gov Identifiers: NCT00005883, NCT00691132, NCT01790204). Due to the potential cancer preventive and therapeutic benefits of PEITC and other isothiocyanates, both biological evaluation and chemical synthesis of these unique compounds have attracted more interest [9,13].

Apart from their potential anticancer effect, isothiocyanates are also valuable intermediates to obtain thioureas. The unique pharmacological properties and structural features of isothiocyanates attract the interest of chemists, and a number of their synthetic routes have been developed. The reported methods include using toxic thiophosgene [14], or less toxic but expensive thiophosgene surrogate reagents such as 1,1′-thiocarbonyldiimidazole [15], to incorporate one carbon atom and one sulfur atom. The alternative incorporation of these two atoms employs carbon disulfide through the decomposition of the in situ-formed dithiocarbamate salt intermediates [16,17,18]. In this strategy, several reagents have been reported, for example, dicyclohexylcarbodiimide [16], 2-chloro-1,3-dimethylimidazo-liniumchloride [17], and the more recently reported di-tert-butyl dicarbonate [18] and tosyl chloride [19]. It is of importance to develop novel and potent isothiocyanates via the optimization of PEITC, while few analogues of PEITC have shown outstanding anticancer activities regarding their in vitro IC_50_ values. In this paper, we report a concise method to prepare isothiocyanates and conduct a biological evaluation of these compounds. Finally, we find that compound **7** showed the best anti-proliferative activity via modulating the increase of intracellular ROS levels.

## 2. Results and Discussion

### 2.1. Chemistry

**Scheme 1:**
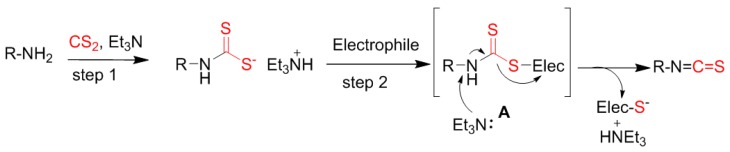
The potential mechanism for the synthesis of isothiocyanates.

During our initial study, the preparation of polar isothiocyanates using tosyl chloride did not encounter any problems. However, in the synthesis of less polar isothiocyanates, a slight excess of tosyl chloride was always difficult to remove from the products by flash chromatography. This problem hampered our progress in obtaining a variety of compounds, thus it is of high interest to find an alternative concise method of doing so. To our best knowledge, there is not yet a generally conceptualized mechanism for the decomposition of dithiocarbamic acid salt mediated by the previously reported reagents. Clearly, the in situ formed dithiocarbamate salt is a potential nucleophile which could react with a nucleophilic receptor, i.e., an electrophile, to form an intermediate A (Scheme 1). Mediated by bases such as triethylamine, an isothiocyanate can be formed after one molecular hydrogen sulfide is eliminated from A and transported to the electrophile. Thus, we envisioned that some electrophiles might be able to mediate the production of isothiocyanates (Scheme 1).

To validate our hypothesis that other electrophiles can replace tosyl chloride, a series of conventional reagents including acetyl chloride (AcCl), tosyl chloride (TsCl), dimethyldichlorosilane (SiMe_2_Cl_2_), benzoyl chloride (BzCl) and sulfonyl chloride (SO_2_Cl_2_) were screened for the synthesis of PEITC from phenethylamine (Table 1). Indeed, all reagents resulted in the expected product in modest to good yields, ensuring that the decomposition of the dithiocarbmate was mediated by its nucleophilic attack on an oncoming electrophile in step 2 (Scheme 1). While the excess tosyl chloride cannot be cleared after the reaction work-up, causing an issue with the purification of low polar isothiocyanates by flash chromatography, acetyl chloride could avoid such a problem and also provided excellent yields. Thus, acetyl chloride was employed to prepare various isothiocyanates in this current work. It is noteworthy that analytic grade tetrahydrofuran (THF) and triethylamine might be used in the late synthesis of some isothiocyanates although the reactions were performed under anhydrous conditions in the screening study. It was also found that triethylamine could be replaced by inorganic bases such as potassium carbonate (data not shown).

**Figure 1:**
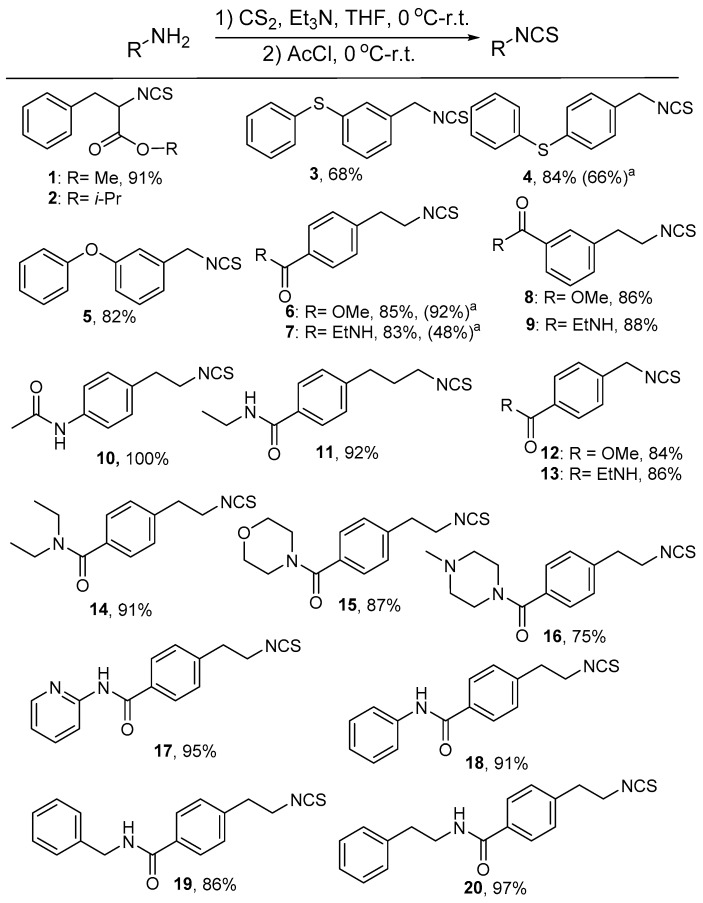
Structures of isothiocyanates and their preparation using acetyl chloride. Note: Unless otherwise stated, all the yields are isolated yields. ^a^ The number in the brackets indicates the yield when prepared with tosyl chloride.

During our study, the preparation of isothiocyanates **1** and **2** actually did not encounter any problems when tosyl chloride was applied. However, the purification of the low polar isothiocyanates **3**–**5** encountered difficulty in the full removal of excess tosyl chloride from the compounds. This problem was successfully avoided when acetyl chloride was employed (Figure 1). The remaining isothiocyanates **6**–**20** were also easily prepared at excellent yields, similar to or better than the yields obtained with tosyl chloride used, as exemplified in the synthesis of **4**, **6** and **7**.

### 2.2. Biology

To design isothiocyanates with better bioactivity is a challenge, because the mechanism of their inhibition of tumor cell growth is still debated and their exact targets remain unclear. Based on our experience, we envisioned that aromatic rings and a chain with reasonable length might be essential to maintain bioactivity. The substituents on the aromatic ring, varying in space size and chemical properties, might influence the compound’s selectivity to bind to certain targets. Thus, isothiocyanates **1** and **2** (Figure 1) were firstly designed and they were prepared from a phenylalanine. Then, **3**–**5** were obtained, identified by another benzene ring tethered through an oxygen or sulfur atom on either the *meta*- or *para-*position of PEITC. Such structural variation would justify whether a bulky group could increase its selectivity or binding affinity to certain targets. Furthermore, compounds **6** and **7** were designed and synthesized because of the possibility that such functional amides and esters might result in an improvement of PEITC bioavailability and the formation of potential hydrogen bonds with some potential targets.

In our preliminary study, isothiocyanates **1**–**7** were screened against two types of malignant cancer cell lines, Panc 1 (pancreatic cancer) and HGC 27 (gastric cancer), since these pancreatic and gastric cancers are fatal and have a poor prognosis (Table 2) [20,21,22,23].

Compared to PEITC, the new synthetic isothiocyanates **1** and **2** showed worse or similar bioactivity especially against Panc 1, implying that extra carboxylate function groups next to the isothiocyanate do not contribute to the biological activity of PEITC. In isothiocyanates **3**–**5**, additional bulky benzene ring did not increase their anticancer activity much compared to PEITC. However, the carboxylate functional groups in isothiocyanates **6** and **7** substantially improved their ability to kill cancer cells. In particular, compound **7** with the *para* amide on the benzene ring of PEITC showed the best potency with 5 or 6 fold increasement. Based on the structure of **7**, the subsequent isothiocyanates **8**–**20** were designed and prepared in order to study their structure and activity relationship. Among the designed compounds, the carboxylate functional groups remained unchanged because they might contribute to the increment of inhibitory effects, likely by either increasing bioavailability or by forming hydrogen bonds with potential targeted macromolecules.

Compounds **8** and **9** with respective meta-positioned ester and amide functional groups showed a slightly lower anticancer activity compared to **6** and **7**, implying that substituents on the para-position was better. Compound **10,** having an acetamide replace the ethyl amide in **7,** also showed similar potency to **7**, further confirming the importance of the carboxylate function groups in the para-position. The length variation of chains linking the benzene ring and isothiocyanate function group provided compounds **11**–**13**. The subsequent biological evaluation demonstrated that a longer or shorter chain of **7** diminished its potency, while a two-carbon linker provided the best potency. This finding implied that the chain length might be crucial for maintaining a balance between rigidity and flexibility to gain more potency. Thus, more structural modifications were performed on the skeleton of **7**; varying the amine motif provided compounds **14**–**20**, with the ethyl amine in **7** replaced by diethyl amine, morpholine, piperazine, pyridine-2-amine, aniline, and other amines (Figure 1). The variation of the amine motif in **7** did not gain more potency to inhibit cancer growth in the newly prepared compounds **14**–**20**. Thus, compound **7** was selected for further biological study, using PEITC and **6** for comparison.

**Figure 2:**
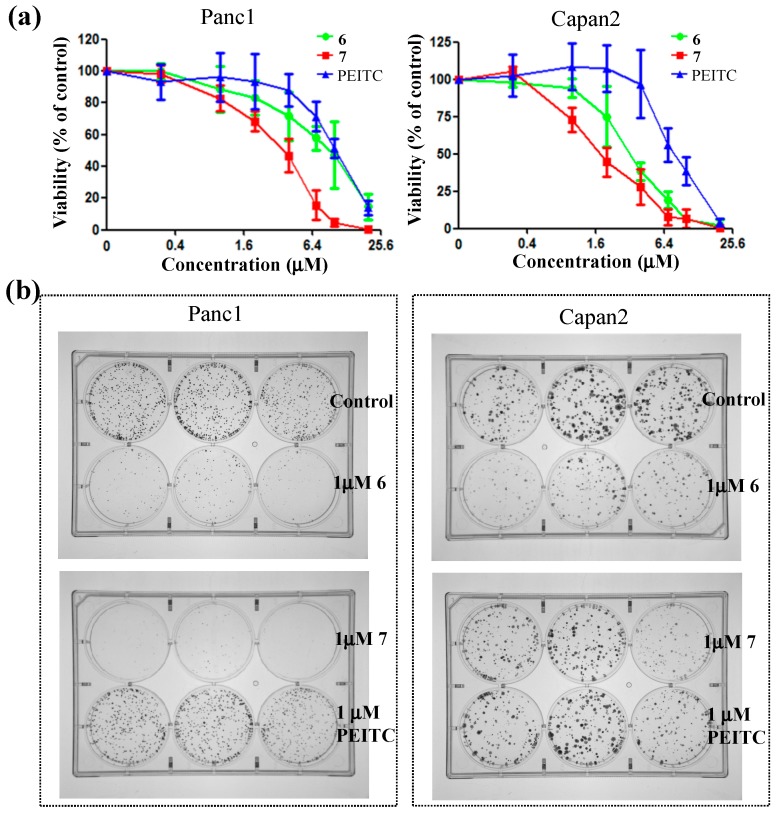
The cellular toxicity of **6** and **7** on the human pancreatic cancer cell lines Panc1 and Capan2. (**a**) MTS assay after 72 h treatment; (**b**) 14-day colony formation (triplicate wells).

**Figure 3:**
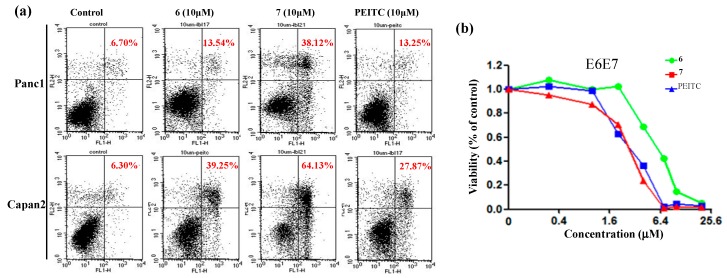
(**a**) The apoptosis induction caused by **6** and **7** in pancreatic cancer cells. Percentage numbers show annexin-V/PI positive cell fraction; (**b**) E6E7 cells were treated with various agents for 72 h before MTS assay.

Pancreatic cancer is one of the most fatal human cancers which remains a challenging health problem due to its high metastases [24]. Our further biological evaluation of compound **7** focused on the human pancreatic cancer cell lines Panc1 and Capan2 (Figure 2). Compound **7** showed better anti-proliferative capability in a dose-dependent manner compared to PEITC and **6**, as shown in Figure 2a. In the colony formation experiments, **7** substantially inhibited Panc1 growth even at 1 μM concentration (Figure 2b). Compared to PEITC, **6** and **7** induced higher apoptosis of Panc1 and Capan2 after 72 h treatment at 10 μM concentration (Figure 3a). However, **6** and **7** caused similar or less cytotoxicity to non-cancerous cells, as evidenced by the cell viability of E6E7 that is an immortalized human pancreatic ductal epithelium cell line (Figure 3b).

**Figure 4:**
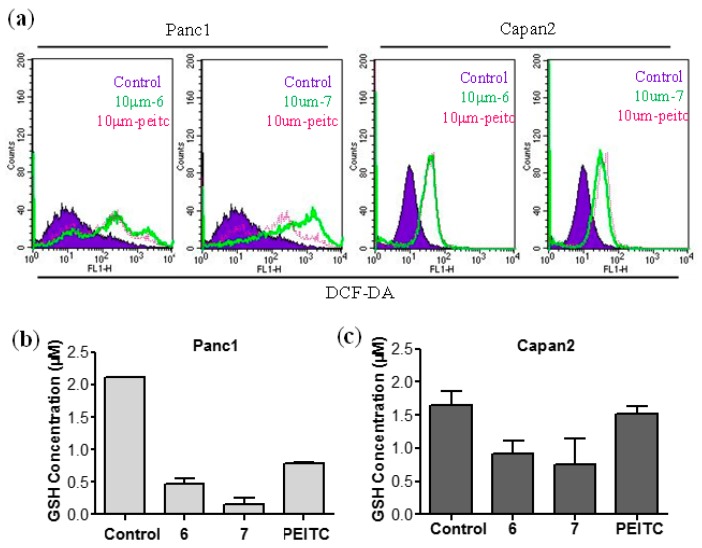
Compounds **6** and **7** induced a redox imbalance in pancreatic cancer cells. (**a**) The content of hydrogen peroxide (6 h); (**b**,**c**) the content of cellular GSH in Panc1 (**b**) and Capan2 (**c**) treated for 2 h with compounds at a concentration of 10 μM.

ROS play important roles in the regulation of cell survival and cell death. In general, a moderate ROS level promotes cell proliferation, whereas an excessive increase of ROS can induce cell death by causing cellular redox imbalance [25,26]. Since PEITC killed malignant cancer cells via the ROS modulation pathway, the effect of **6** and 7 on intracellular ROS levels was finally tested. Indeed, **7** significantly increased the ROS level as PEITC, as shown in Figure 4a. Intracellular glutathione (GSH) is a very important endogenous antioxidant to defend high oxidative stress in malignant cancer cells [27,28]. Thus, we also tested whether our synthetic isothiocyanates **6** and **7** could deplete GSH in Panc1 and Capan2 cells (Figure 4b,c). As expected, GSH depletion by **7** was the most significant, followed by **6** and PEITC, consistent to their anticancer activity.

## 3. Materials and Methods

### 3.1. General Procedure Exemplified by the Synthesis of PEITC

All reactions were performed under argon, unless indicated otherwise. Column chromatography was performed using silca gel (200–300 mesh). NMR spectra were recorded on a Bruker 400 MHz spectrometer (Rheinstetten, Germany). ^1^H-NMR spectra were referenced internally to either CDCl_3_ (δ 7.26 ppm) or DMSO*-d*_6_ (δ 2.50 ppm). ^13^C-NMR spectra were referenced to CDCl_3_ (δ 77.16 ppm, the middle peak) or DMSO*-d*_6_ (δ 39.52 ppm, the middle peak). Chemical shifts (δ) were reported as part per million (ppm), and coupling constants (*J*) reported in Hertz (Hz). Mass spectra were recorded with an Agilent LC-MS 6120 spectrometer (Santa Clara, CA, USA). High-resolution mass spectrometry (HRMS) was carried out using a Shimadzu LCMS-IT-TOF spectrometer (Kyoto, Japan). ^1^H-NMR spectrum and ^13^C-NMR spectrum can be found at Supplementary Materials.

To a solution of phenethylamine (200 mg, 1.65 mmol) and Et_3_N (0.64 mL, 4.95 mmol) in anhydrous THF (2.5 mL) cooled with an ice bath, a solution of CS_2_ (0.12 mL, 1.98 mmol) was slowly dropped in. The reaction solution was stirred at room temperature for 0.5 h, after which AcCl (0.14 mL, 1.98 mmol) was dropped in at 0 °C, and after 5 min the mixture was warmed to room temperature for 15–30 min. When the starting amine was finished, as verified by checking thin layer chromatography (T.L.C.), 1 M HCl (aq., 2 mL) was added to quench the reaction. The solution was extracted by EtOAc three times. All organic phases were combined and washed with brine, dried over Na_2_SO_4_, and finally filtered and concentrated under reduced pressure. The residue was purified by column chromatography on silica gel (EtOAc/petroleum = 1/1) to provide phenethyl isothiocyanate (PEITC) (253 mg, 94%).

### 3.2. Characterization of Synthetic Isothiocyanates

Phenethyl isothiocyanate/PEITC. Yellow liquid, yield: 94%, ^1^H-NMR (400 Hz, CDCl_3_): δ 7.25–7.40 (m, 5H), 3.75 (t, 2H, *J* = 6.8 Hz), 3.02 (t, 2H, *J* = 6.8 Hz).

*Methyl 2-isothiocyanato-3-phenylpropanoate* (**1**). Pale yellow liquid, yield: 91%, ^1^H-NMR (400 Hz, CDCl_3_): δ 7.30–7.38 (m, 3H), 7.21–7.26 (m, 2H), 4.48 (dd, 1H, *J* = 4.8, 8.0 Hz), 3.81 (s, 3H), 3.25 (dd, 1H, *J* = 4.8, 14.0 Hz), 3.13 (dd, 1H, *J* = 8.0, 14.0 Hz).

*Isopropyl 2-isothiocyanato-3-phenylpropanoate* (**2**). Yellow liquid, yield: 87%, ^1^H-NMR (400Hz, CDCl_3_): δ 7.22–7.35 (m, 5H), 5.06 (m, 1H), 4.40 (dd, 1H, *J* = 5.2, 8.0 Hz), 3.22 (dd, 1H, *J* = 4.8, 13.6 Hz), 3.12 (dd, 1H, *J* = 8.0, 13.6 Hz), 1.26 (s, 3H), 1.25 (S, 3H); ^13^C-NMR (100 Hz, CDCl_3_): δ 167.5, 135.3, 129.5, 128.8, 127.7, 70.8, 61.0, 39.8, 21.8. HRMS calcd for C_13_H_14_NO_2_S^−^: 248.0751, found 248.0747.

*(4-(Isothiocyanatomethyl)phenyl)(phenyl)sulfane* (**3**). Pale yellow liquid, yield: 84%, ^1^H-NMR (400 Hz, CDCl_3_): δ 7.14–7.32 (m, 9H), 4.59 (s, 2H) ppm; ^13^C-NMR (100 Hz, CDCl_3_): δ 137.2, 134.9, 133.0 132.0 130.9, 129.5, 127.8, 127.7, 48.5 ppm. HRMS calcd for C_14_H_10_NS_2_^−^: 256.0260, found 256.0266.

*(3-(Isothiocyanatomethyl)phenyl)(phenyl)sulfane* (**4**). Yellow liquid, yield: 68%, ^1^H-NMR (400 Hz, CDCl_3_): δ 7.44–7.19 (m, 9H), 4.68 (s, 2H) ppm; ^13^C-NMR (100 Hz, CDCl_3_): δ 137.9, 135.5, 132.1, 130.1, 129.8, 129.6, 129.1, 128.4, 127.9, 127.0, 125.2, 48.5 ppm. HRMS calcd for C_14_H_10_NS_2_^−^: 256.0260, found 256.0255.

*1-(Isothiocyanatomethyl)-3-phenoxybenzene* (**5**). Pale yellow liquid, yield: 82%, ^1^H-NMR (400 Hz, CDCl_3_): δ 7.32–7.39 (m, 3H), 7.15 (t, 1 H, *J* = 7.2 Hz), 6.96–7.06 (m, 5H), 4.67 (s, 2H) ppm; ^13^C-NMR (100 Hz, CDCl_3_): δ 158.1, 156.7, 136.3, 130.5, 130.0, 123.9, 121.5, 119.4, 118.5, 117.1, 48.5 ppm.

*Methyl4-(2-isothiocyanatoethyl)benzoate* (**6**). White solid, yield: 85%, ^1^H-NMR (400 Hz, CDCl_3_): δ 8.01 (d, 2H, *J* = 8.0 Hz), 7.29 (d, 2H, *J* = 8.0 Hz), 3.91 (s, 3H), 3.75 (t, 2H, *J* = 6.8 Hz), 3.03 (t, 2H, *J* = 6.8 Hz) ppm; ^13^C-NMR (100 Hz, CDCl_3_): δ 166.9, 142.4, 130.2, 129.3, 129.0, 52.2, 46.0, 36.5 ppm; m.p. 119.9–123.1 °C; MS (ESI, *m*/*z*) 222.0 (M + H^+^).

*N-Ethyl-4-(2-isothiocyanatoethyl)benzamide* (**7**): White solid, yield: 83%, ^1^H-NMR (400 Hz, CDCl_3_): δ 7.67 (d, 2H, *J* = 8.0 Hz), 7.21 (d, 2H, *J* = 8.0 Hz), 6.05 (brs, 1H), 3.68 (t, 2H, *J* = 6.4 Hz), 3.43 (t, 2H, *J* = 6.4 Hz), 2.96 (t, 2H, *J* = 6.4 Hz), 1.18 (t, 3H, *J* = 7.2 Hz) ppm; ^13^C-NMR (100 Hz, CDCl_3_): δ 167.1, 140.6, 134.0, 129.1, 127.5, 46.2, 36.4, 35.1, 15.0 ppm; m.p. 89.8–92.5 °C; HRMS calcd for C_12_H_14_N_2_OSNa^+^: 257.0719, found 257.0722.

*Methyl 3-(2-isothiocyanatoethyl)benzoate* (**8**). White solid, yield: 86%, ^1^H-NMR (400 Hz, CDCl_3_): δ 7.96–7.94 (m, 1H), 7.90 (s, 1H), 7.42 (d, 2H, *J* = 4.4 Hz), 3.92 (s, 3H), 3.76 (t, 2H, *J* = 6.8 Hz), 3.04 (t, 2H, *J* = 6.8 Hz); ^13^C-NMR (100 Hz, CDCl_3_): δ 166.9, 137.5, 133.6, 130.8, 129.9, 129.0, 128.6, 52.3, 46.2, 36.3 ppm; m.p. 86.5–90.8 °C; HRMS calcd for C_11_H_11_NO_2_SNa^+^: 244.0403, found 244.0397.

*N-Ethyl-3-(2-isothiocyanatoethyl)benzamide* (**9**). Yellow solid, yield: 88%, ^1^H-NMR (400 Hz, CDCl_3_): δ 7.62–7.67 (m, 1H), 7.62 (s, 1H), 7.27–7.32 (m, 2H), 6.51 (brs, 1H), 3.65 (dd, 2H, *J* = 11.2, 6.4 Hz), 3.34–3.52 (m, 2H), 2.93 (dd, 2H*, J* = 11.2, 6.4 Hz), 1.17 (t, 3H, *J* = 8.0 Hz); ^13^C-NMR (100 Hz, CDCl_3_): δ 167.4, 137.5, 135.6, 131.8, 129.1, 127.6, 126.0, 46.3, 36.4, 35.1, 14.9. HRMS calcd for C_12_H_14_N_2_OSNa^+^: 257.0719, found 257.0714.

*N-(4-(2-Isothiocyanatoethyl)phenyl)acetamide* (**10**). White solid, yield: 100%, ^1^H-NMR (400 Hz, DMSO*-d*_6_): δ 9.90 (brs, 1H), 7.52 (d, 2H, *J* = 8.2 Hz), 7.19 (d, 2H, *J* = 8.2 Hz), 3.86 (t, 2H, *J* = 6.4 Hz), 2.89 (t, 2H, *J* = 6.4 Hz), 2.03 (s, 3H) ppm; ^13^C-NMR (100 Hz, DMSO*-d*_6_): δ 168.1, 138.0, 132.0, 129.0, 119.0, 46.0, 34.7, 23.9 ppm; m.p. 154.0–158.1 °C; MS (ESI, *m/z*) 221.1 (M + H^+^), 441.1 (2M + H^+^); HRMS calcd for C_11_H_11_N_2_OS^−^: 219.0598, found 219.0602.

*N-Ethyl-4-(3-isothiocyanatopropyl)benzamide* (**11**). Colorless oil, yield: 92%, ^1^H-NMR (400 Hz, CDCl_3_): δ 7.71 (d, 2H, *J* = 8.0 Hz), 7.25 (d, 2H, *J* = 8.0 Hz), 6.10 (brs, 1H), 3.53–3.46 (m, 4H), 2.80 (t, 2H, *J* = 7.2 Hz), 1.99–2.06 (m, 2H), 1.25 (t, 3H, *J* = 7.2 Hz) ppm; ^13^C-NMR (100 Hz, CDCl_3_): δ 167.4, 143.7, 133.1, 128.7, 127.4, 44.2, 35.0, 32.5, 31.2, 15.0 ppm. MS (ESI, *m*/*z*) 249.1 (M + H^+^), 497.2 (2M + H^+^); HRMS calcd for C_13_H_15_N_2_OS^−^: 247.0911, found 247.0914.

*Methyl 4-(isothiocyanatomethyl)benzoate* (**12**). Brown liquid, yield: 84%, ^1^H-NMR (400 Hz, CDCl_3_): δ 8.05 (d, 2H, *J* = 8.0 Hz), 7.38 (d, 2H, *J* = 8.0 Hz), 4.77 (s, 2H), 3.91 (s, 3H) ppm; ^13^C-NMR (100 Hz, CDCl_3_): δ 166.5, 139.3, 130.3, 126.8, 52.33, 48.5 ppm.

*N-Ethyl-4-(isothiocyanatomethyl)benzamide* (**13**). White solid, yield: 86%, ^1^H-NMR (400 Hz, CDCl_3_): δ 7.90 (d, 2H, *J* = 8.4 Hz), 7.37 (d, 2H, *J* = 8.4 Hz), 6.14 (brs, 1H), 4.76 (s, 2H), 3.54–3.47 (m, 2H), 1.26 (t, 3H, *J* = 7.2 Hz); ^13^C-NMR (100 Hz, CDCl_3_): δ 166.8, 137.6, 135.0, 127.7, 127.0, 48.5, 35.1, 15.0 ppm; m.p. 94.6–98.7 °C; HRMS calcd for C_11_H_11_N_2_OS^−^: 219.0598, found 219.0592.

*N,N-Diethyl-4-(2-isothiocyanatoethyl)benzamide* (**14**). Pale brown liquid, yield: 91%, ^1^H-NMR (400 Hz, CDCl_3_): δ 7.32–7.34 (d, 2H, *J* = 8.0 Hz), 7.22–7.24 (d, 2H, *J* = 8.0 Hz), 3.72 (t, 2H, *J* = 6.8 Hz), 3.52 (brs, 2H), 3.24 (brs, 2H), 2.99 (t, 2H, *J* = 6.8 Hz), 1.10–1.24 (m, 6H) ppm; ^13^C-NMR (100 Hz, CDCl_3_): δ 171.1, 138.2, 136.3, 129.0, 126.9, 46.2, 43.4, 39.4, 36.3, 28.5, 14.3, 13.0 ppm. HRMS calcd for C_14_H_19_N_2_OS^+^: 263.1213, found 263.1200.

*(4-(2-Isothiocyanatoethyl)phenyl)(morpholino)methanone* (**15**). Yellow liquid, yield: 87%, ^1^H-NMR (400 Hz, CDCl_3_): δ 7.38 (d, 2H, *J* = 8.0 Hz), 7.26 (d, 2H, *J* = 8.0 Hz), 3.73 (t, 2H, *J* = 6.4 Hz), 3.44–3.76 (m, 8H), 3.00 (t, 2H, *J* = 6.4 Hz) ppm; ^13^C-NMR (100 Hz, CDCl_3_): δ 170.2, 139.1, 134.5, 129.1, 127.8, 67.0, 60.4, 46.2, 36.3 ppm. MS (ESI, *m/z*) 277.2 (M + H^+^). HRMS calcd for C_14_H_16_N_2_O_2_SNa^+^: 299.0825, found 299.0832.

*(4-(2-Isothiocyanatoethyl)phenyl)(4-methylpiperazin-1-yl)methanone* (**16**). Brown solid, yield: 80%, ^1^H-NMR (400 Hz, CDCl_3_): δ 7.30–7.16 (m, 4H), 3.70–3.38 (m, 4H), 3.65 (t, 2H, *J* = 6.8 Hz), 2.92 (t, 2H, *J* = 6.8 Hz), 2.41–2.36 (m, 4H), 2.24 (s, 3H); ^13^C-NMR (100 Hz, CDCl_3_): δ 170.2, 139.0, 134.8, 129.1, 127.8, 55.2, 46.2, 46.0, 36.4, 21.2 ppm; HRMS calcd for C_15_H_20_N_3_OS^+^: 290.1322, found 290.1317.

*4-(2-Isothiocyanatoethyl)-N-(pyridin-2-yl)benzamide* (**17**). White solid, yield: 95%, ^1^H-NMR (400 Hz, CDCl_3_): δ 8.66 (s, 1H), 8.38 (d, 1H, *J* = 8.4 Hz), 8.29 (d, 1H, *J* = 4.0 Hz), 7.92 (d, 2H, *J* = 8.0 Hz), 7.74–7.78 (m, 1H), 7.36 (d, 2H, *J* = 8.0 Hz), 7.06–7.09 (m, 1H), 3.78 (t, 2H, *J* = 7.2 Hz), 3.07 (t, 2H, *J* = 7.2 Hz) ppm; ^13^C-NMR (100 Hz, CDCl_3_): δ 151.6, 148.1, 141.8, 138.7, 133.4, 129.5, 127.9, 120.1, 114.3, 46.1, 36.5, 29.8 ppm; m.p. 139.6–141.3 °C; MS (ESI, *m/z*) 284.1 (M + H^+^). HRMS calcd for C_15_H_12_N_3_OS^−^: 282.0707, found 282.0697.

*4-(2-Isothiocyanatoethyl)-N-phenylbenzamide* (**18**). White solid, yield: 91%, ^1^H-NMR (400 Hz, CDCl_3_): δ 7.85 (d, 2H, *J* = 8.0 Hz), 7.64 (d, 2H, *J* = 8.0 Hz), 7.39–7.33 (m, 4H), 7.14–7.18 (m, 1H), 3.77 (t, 2H, *J* = 6.8 Hz), 3.06 (t, 2H, *J* = 6.8 Hz); ^13^C-NMR (100 Hz, CDCl_3_): δ 165.5, 141.3, 138.0, 134.1, 129.4, 129.2, 127.7, 124.8, 120.4, 46.1, 36.4 ppm; m.p. 137.0–142.9 °C; HRMS calcd for C_16_H_14_N_2_OSNa^+^: 305.0719, found 305.0707.

*N-Benzyl-4-(2-isothiocyanatoethyl)benzamide* (**19**). White solid, yield: 86%, ^1^H-NMR (400 Hz, CDCl_3_): δ 7.67 (d, 2H, *J* = 8.0 Hz), 7.25–7.16 (m, 7H), 6.39 (brs, 1H), 4.54 (d, 2H, *J* = 5.6 Hz), 3.64 (t, 2H, *J* = 6.8 Hz), 2.92 (t, 2H, *J* = 6.8 Hz); ^13^C-NMR (100 Hz, CDCl_3_): δ 167.1, 140.9, 138.3, 133.5, 129.2, 128.9, 128.0, 127.8, 127.6, 46.1, 44.3, 36.4 ppm; m.p. 143.0–145.8 ^°^C; HRMS calcd for C_17_H_16_N_2_OSNa^+^: 319.0876, found 319.0877.

*4-(2-Isothiocyanatoethyl)-N-phenethylbenzamide* (**20**). Yellow solid, yield: 97%, ^1^H-NMR (400 Hz, CDCl_3_): δ 7.58 (d, 2H, *J* = 8.0 Hz), 7.23 (d, 2H, *J* = 6.8 Hz), 7.18–7.14 (m, 5H), 6.12 (brs, 1H), 3.66–3.60 (m, 4H), 2.92 (t, 2H, *J* = 6.8 Hz), 2.85 (t, 2H, *J* = 6.8 Hz); ^13^C-NMR (100 Hz, CDCl_3_): δ 167.2, 140.8, 139.0, 133.8, 129.1, 128.9, 128.8, 127.5, 126.7, 46.1, 41.3, 36.4, 35.8 ppm; m.p. 98.2–102.5 °C; HRMS calcd for C_18_H_18_N_2_OSNa^+^: 333.1032, found 333.1046.

### 3.3. Biological Cellular Assay

3-(4,5-Dimethylthiazol-2-yl)-5-(3-carboxymethoxyphenyl)-2-(4-sulfophenyl)-2H-tetrazolium (MTS) powder and DMSO were bought from Sigma. DMEM medium, RPMI-1640 medium and fetal bovine serums were purchased from GIBCO.

#### 3.3.1. Cell Culture

Human cancer cell lines Panc1 and Capan2 (pancreatic cancer), HGC27 (gastric cancer), and E6E7 were obtained from Sun-Yat Sen University Cancer Center. Cells were cultured in medium supplemented with 5–10% fetal bovine serum under the conditions of a humidified 5% carbon dioxide incubator. Every two to three days the medium was removed, when the confluence of cells reached about 80%, and trypsin was used to digest the cells.

#### 3.3.2. MTS Assay

Human cancer cells in logarithmic phase were inoculated in a 96-well plate (three wells for each drug concentration), with 1000–10,000 cells in each well. Once the cells were adherent, 100 μL medium containing different concentrations of drugs were added, and after incubation for 72 h, 20 μL MTS (5 mg/mL) was added, followed by incubation for another 4 h. At the end of the incubation period, DMSO was used to dissolve purple formazen (the production of formazen was proportional to the number of viable cells). Then, the plate was shaken for 10–15 min and the optical density (OD) value of the plate was detected by microreader (Thermo Scientific, Waltham, USA) at a wavelength of 570 nm. The survival rates were expressed as follows: survival rate % = mean OD value of drug treatment/mean OD value of control × 100% (Experiments were done for three times independently).

#### 3.3.3. Colony Formation

Human cancer cells were seeded in 6-well plates at 500 cells per well. Then, cells were incubated with the indicated agents for 14 days, followed by fixation and staining with crystal violet. The samples were photographed and the colonies were counted.

#### 3.3.4. Apoptosis and ROS Measurement

Apoptosis was detected using Annexin V/PI staining. Cells were harvested and stained with Annexin V and PI for 15 min, and apoptosis rates were analyzed using BD Calibur flow cytometry. Similarly, cells were stained for 30 min with DCF-DA for ROS detection. The samples were washed and analyzed using flow cytometry.

#### 3.3.5. GSH Levels

Cellular GSH contents were measured using a GSH-Glo™ Glutathione Assay kit (Promega, WI, USA) according to the protocol provided by the manufacturer.

## 4. Conclusions

A concise preparation of isothiocyanates mediated by acetyl chloride and other electrophiles were studied. Using acetyl chloride, the expected isothiocyanates were obtained at good to excellent yields and the limitation of the often used tosyl chloride was overcome. These factors along its low cost would make acetyl chloride practical to prepare isothiocyanates. In our study, a series of novel isothiocyanates were designed and conveniently prepared using our newly discovered method. A biological evaluation on two malignant human cancer cell lines, pancreatic cancer Panc1 and gastric cancer HGC27, was investigated. Finally, compound **7** was found to have a better growth inhibition in the test cancer cells relative to PEITC that is one of the most studied naturally occuring isothiocyanates. In further study, compound **7** substantially inhibited the colony formation of pancreatic cancer cell lines Panc1 and Capan2, and caused better apoptosis than PEITC. Compound **7** also induced an increase in the levels of intracellular ROS and depleted the levels of GSH, which is one of the most recognized mechanisms of PEITC. Since several clinical trials with PEITC have been registered, our newly discovered compound **7** could provide therapeutic promise for the treatment of malignant tumors. Further study of the anticancer therapeutic effects of **7** is under way and will be reported in due time.

## Figures and Tables

**Table 1:**
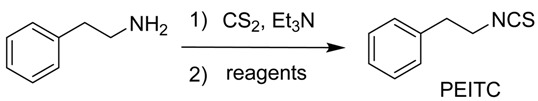
Synthesis of phenethyl isothiocyanate mediated by various reagents.

Entry	1	2	3	4	5
Reagent	AcCl	TsCl	SiMe_2_Cl_2_	BzCl	SO_2_Cl_2_
Yield	94%	70%	79%	42%	80%

*Reagents and Conditions*: (1) phenylethylamine (1 equiv.), CS_2_ (1.2 equiv.), and Et_3_N (3 equiv.) in anhydrous THF, 0 °C–r.t.; (2) reagents (1.2 equiv.) was added, 0 °C–r.t.

**Table 2:** Anti-proliferative activities of isothiocyanates against two types of human cancer cell lines, Panc1 (pancreatic cancer) and HGC27 (gastric cancer), measured by MTS assays.

Compound	IC_50_ (μM)	
	Panc1	HGC27
PEITC	10.80 ± 0.25	2.95 ± 0.41
**1**	15.80 ± 1.06	8.79 ± 0.72
**2**	10.87 ± 0.47	1.49 ± 0.06
**3**	14.24 ± 0.50	2.73 ± 0.29
**4**	6.12 ± 0.13	3.44 ± 0.23
**5**	8.05 ± 0.36	2.67 ± 0.37
**6**	4.55 ± 0.32	1.09 ± 0.07
**7**	2.04 ± 0.21	0.46 ± 0.02
**8**	5.78 ± 0.56	0.74 ± 0.01
**9**	3.52 ± 0.11	0.84 ± 0.06
**10**	2.68 ± 0.21	0.93 ± 0.41
**11**	5.08 ± 0.02	1.47 ± 0.10
**12**	8.92 ± 0.43	0.72 ± 0.21
**13**	5.65 ± 0.16	0.99 ± 0.15
**14**	2.25 ± 0.21	0.62 ± 0.04
**15**	3.76 ± 0.05	1.68 ± 0.00
**16**	6.24 ± 0.13	2.37 ± 0.43
**17**	4.70 ± 0.27	0.77 ± 0.18
**18**	2.78 ± 0.72	0.34 ± 0.00
**19**	2.14 ± 0.79	0.72 ± 0.08
**20**	4.06 ± 0.57	0.82 ± 0.06

## Supplementary Materials

Supplementary data including ^1^H-NMR spectrum and ^13^C-NMR spectrum can be available online.
