# Supplementary material for: New Mild and Simple Approach to Isothiocyanates: A Class of Potent Anticancer Agents
Source: Molecules. 2017 Jun 1;22(6):773. doi: 10.3390/molecules22060773 (PMC6152774; doi:10.3390/molecules22060773)
Supplement: Supplementary file 1 [file molecules-22-00773-s001.pdf]

Supplementary Material

New Mild and Simple Approach to Isothiocyanates: A Class of Potent Anticancer Agents

Bingling Luo [1,2,†], Jiankang Wang [1,2,†], Xiaobing Li [1], Wenhua Lu [1], Jing Yang [1], Yumin Hu [1], Peng Huang [1,2] and Shijun Wen [1,2,*]

[1] Sun Yat-Sen University Cancer Center; State Key Laboratory of Oncology in South China; Collaborative Innovation Center for Cancer Medicine; Sun Yat-Sen University, 651 Dongfeng East Road, Guangzhou 510060, China; luobl@sysucc.org.cn (B.L.); lixiaobing0629@126.com (X.L.); luwenh@sysucc.org.cn (W.L.); yangjing@sysucc.org.cn (J.Y.); huym@sysucc.org.cn (Y.H.); huangpeng@sysucc.org.cn (P.H.)

[2] School of Pharmaceutical Sciences, Sun Yat-sen University, 132 Waihuan East Road, Guangzhou 510006, China; wangjk6@mail2.sysu.edu.cn (J.W.)

* Correspondence: wenshj@sysucc.org.cn; Tel.: +86-20-3994-3091

† The authors contributed equally to this work.

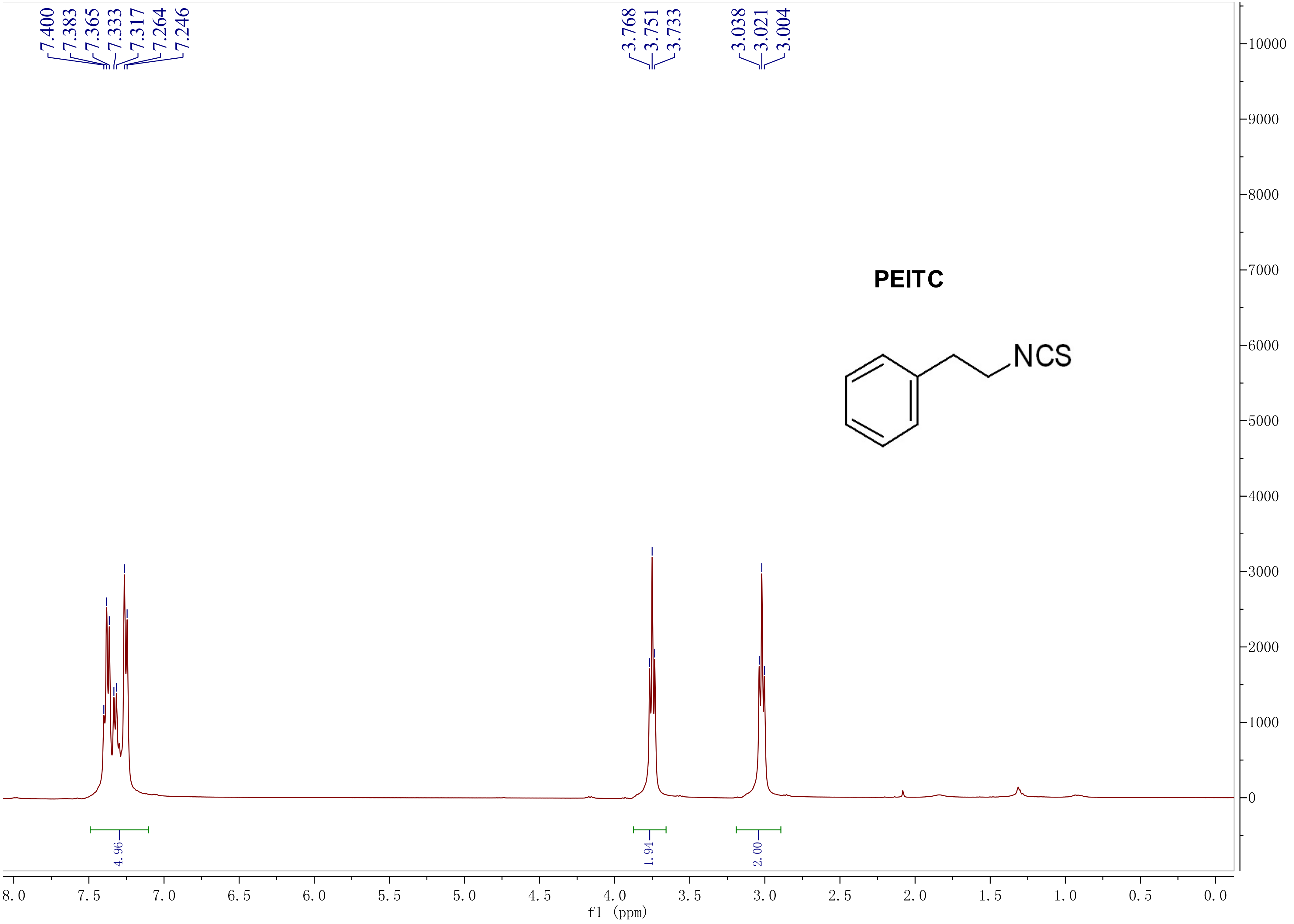
7.400
7.383
7.365
7.333
7.317
7.264
7.246
3.768
3.751
3.733
3.038
3.021
3.004
PEITC
NCS
4.96
1.94
2.00
8.0 7.5 7.0 6.5 6.0 5.5 5.0 4.5 4.0 3.5 3.0 2.5 2.0 1.5 1.0 0.5 0.0
f1 (ppm)
10000 9000 8000 7000 6000 5000 4000 3000 2000 1000 0

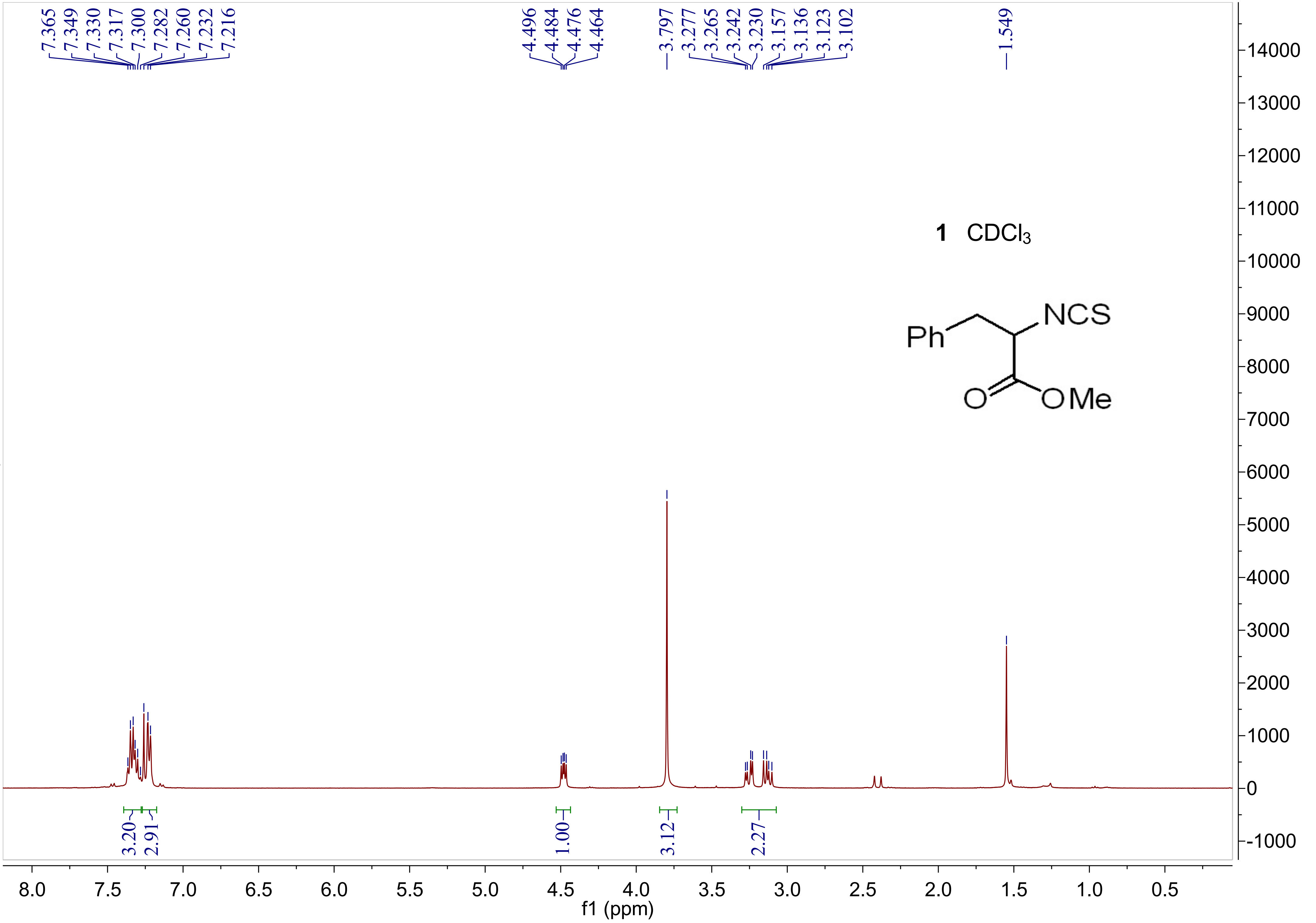
7.365
7.349
7.330
7.317
7.300
7.282
7.260
7.232
7.216
4.496
4.484
4.476
4.464
3.797
3.277
3.265
3.242
3.230
3.157
3.136
3.123
3.102
1.549
1 CDCl3
Ph
NCS
O
OMe
3.20
2.91
1.00
3.12
2.27
f1 (ppm)
8.0
7.5
7.0
6.5
6.0
5.5
5.0
4.5
4.0
3.5
3.0
2.5
2.0
1.5
1.0
0.5
14000
13000
12000
11000
10000
9000
8000
7000
6000
5000
4000
3000
2000
1000
0
-1000

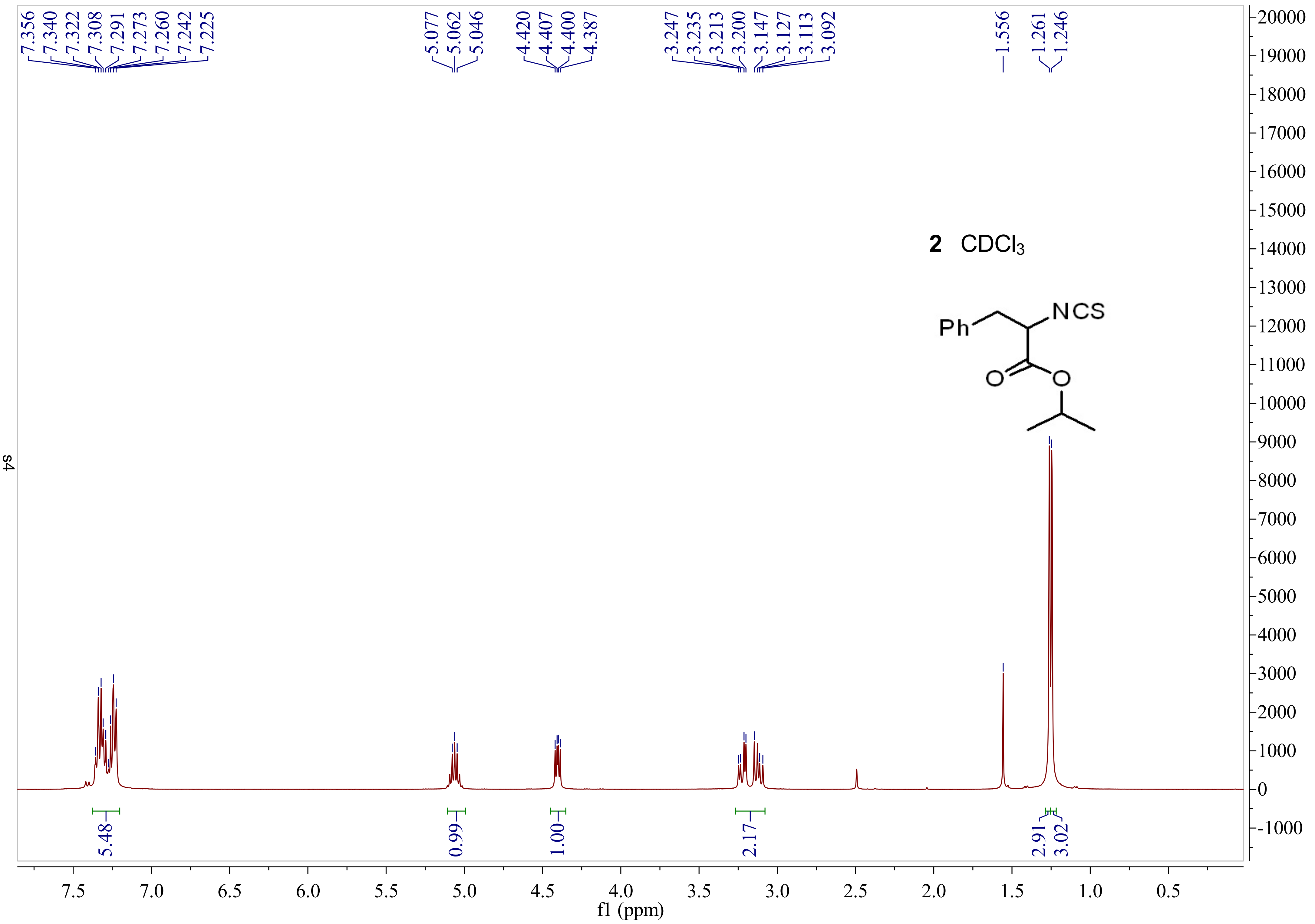

7.356
7.340
7.322
7.308
7.291
7.273
7.260
7.242
7.225
5.077
5.062
5.046
4.420
4.407
4.400
4.387
3.247
3.235
3.213
3.200
3.147
3.127
3.113
3.092
1.556
1.261
1.246
2 CDCl3
Ph
NCS
O
O
5.48
0.99
1.00
2.17
2.91
3.02
fl (ppm)

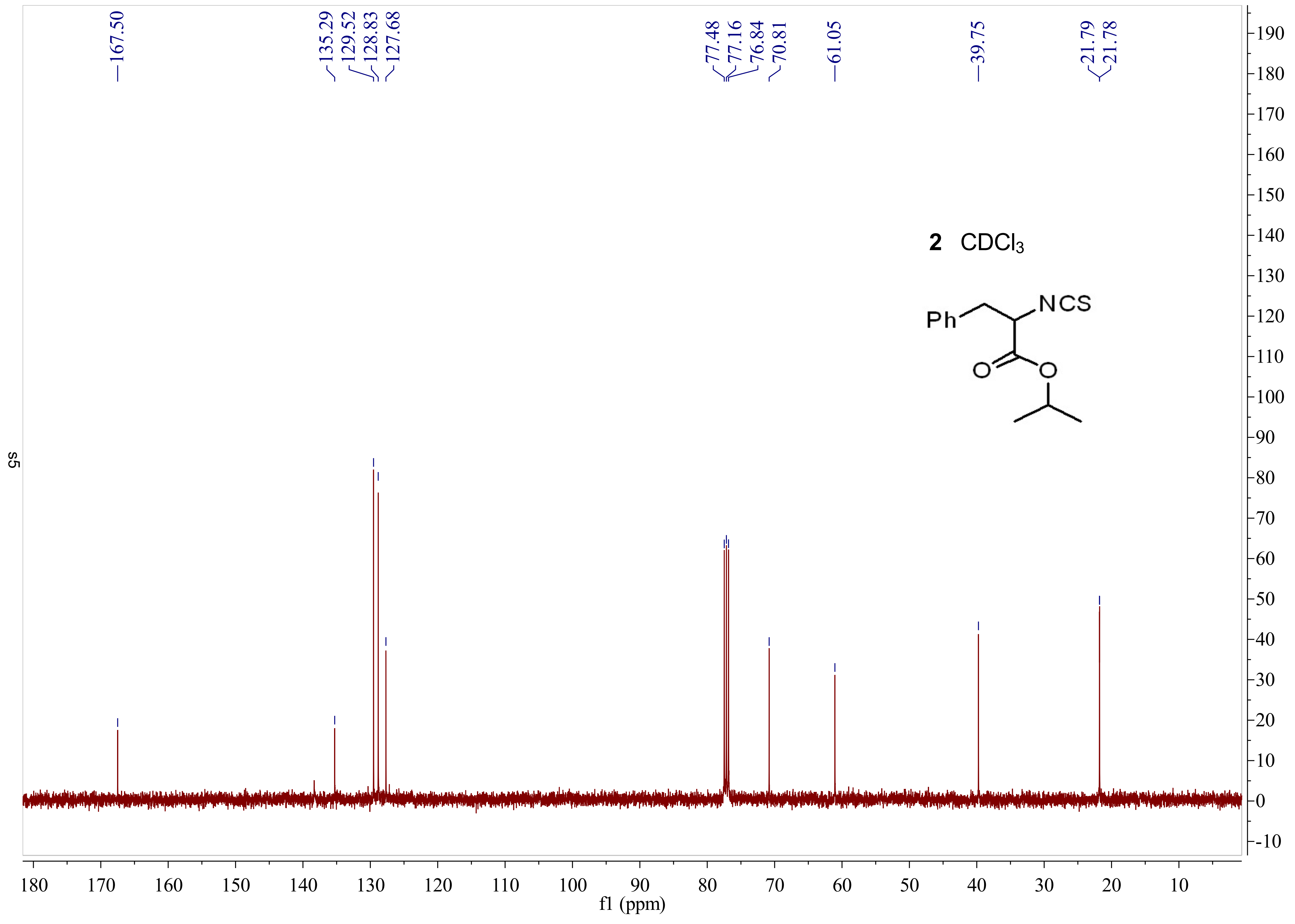

167.50
135.29
129.52
128.83
127.68
77.48
77.16
76.84
70.81
61.05
39.75
21.79
21.78
2 CDCl3
Ph
NCS
O
O
f1 (ppm)

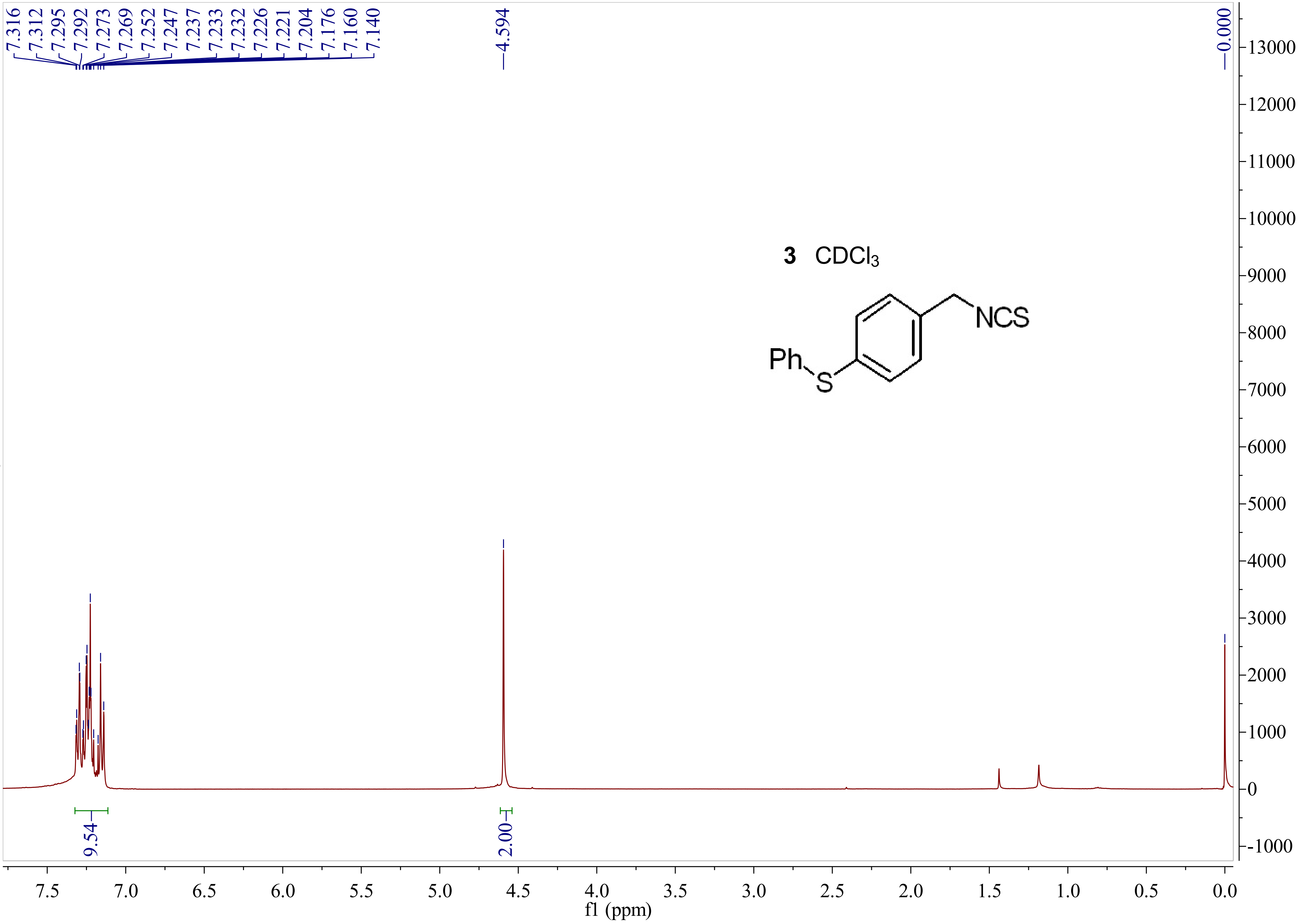
7.316
7.312
7.295
7.292
7.273
7.269
7.252
7.247
7.237
7.233
7.232
7.226
7.221
7.204
7.176
7.160
7.140
4.594
0.000
3 $CDCl_3$
Ph
S
NCS
9.54
2.00
7.5
7.0
6.5
6.0
5.5
5.0
4.5
4.0
3.5
3.0
2.5
2.0
1.5
1.0
0.5
0.0
f1 (ppm)
13000
12000
11000
10000
9000
8000
7000
6000
5000
4000
3000
2000
1000
0
-1000

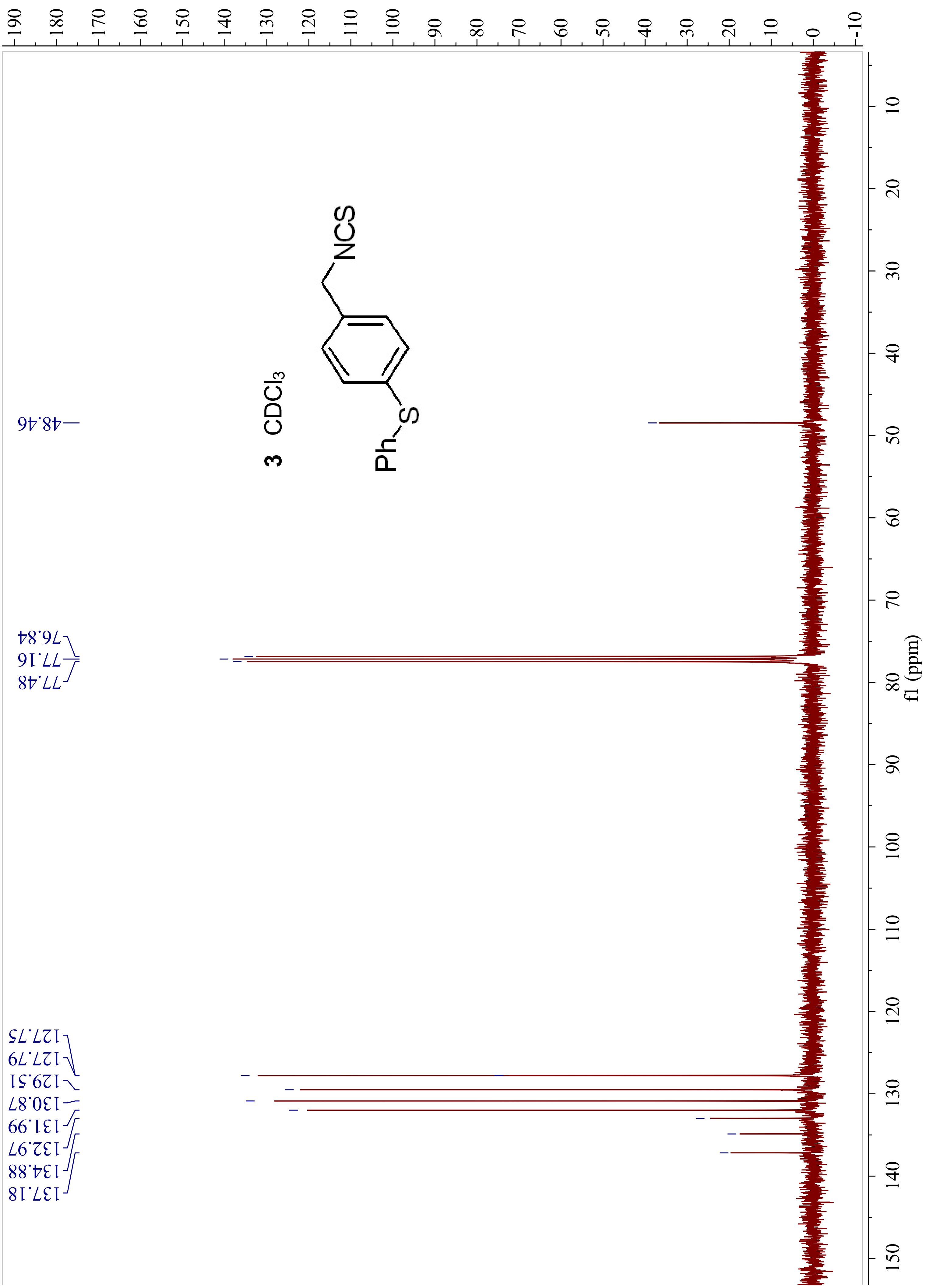
48.46
76.84
77.16
77.48
127.75
127.79
129.51
130.87
131.99
132.97
134.88
137.18
3 CDCl3
Ph
S
NCS
fl (ppm)

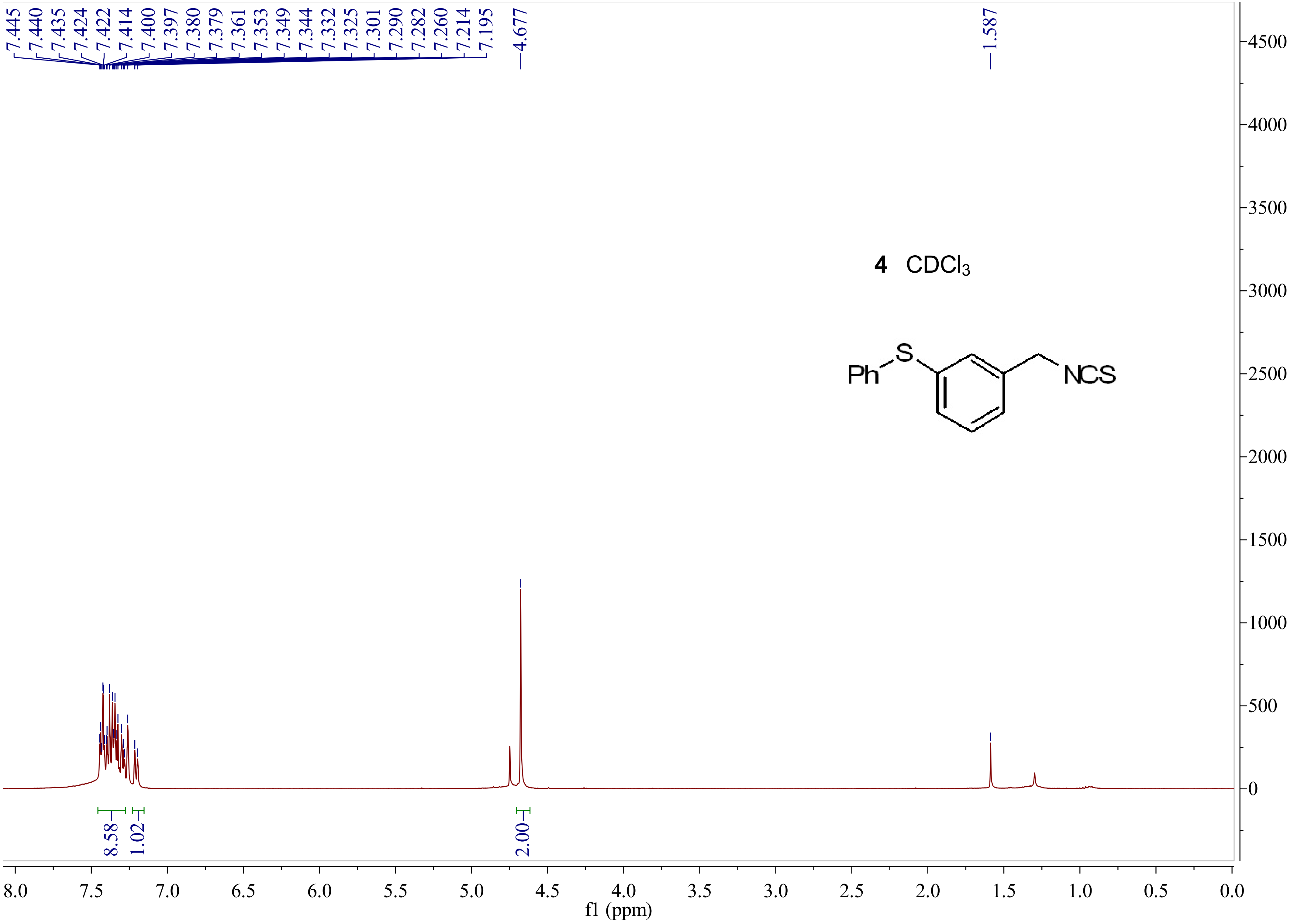

4 CDCl3
Ph S NCS
7.445 7.440 7.435 7.424 7.422 7.414 7.400 7.397 7.380 7.379 7.361 7.353 7.349 7.344 7.332 7.325 7.301 7.290 7.282 7.260 7.214 7.195
4.677
1.587
8.58
1.02
2.00
f1 (ppm)

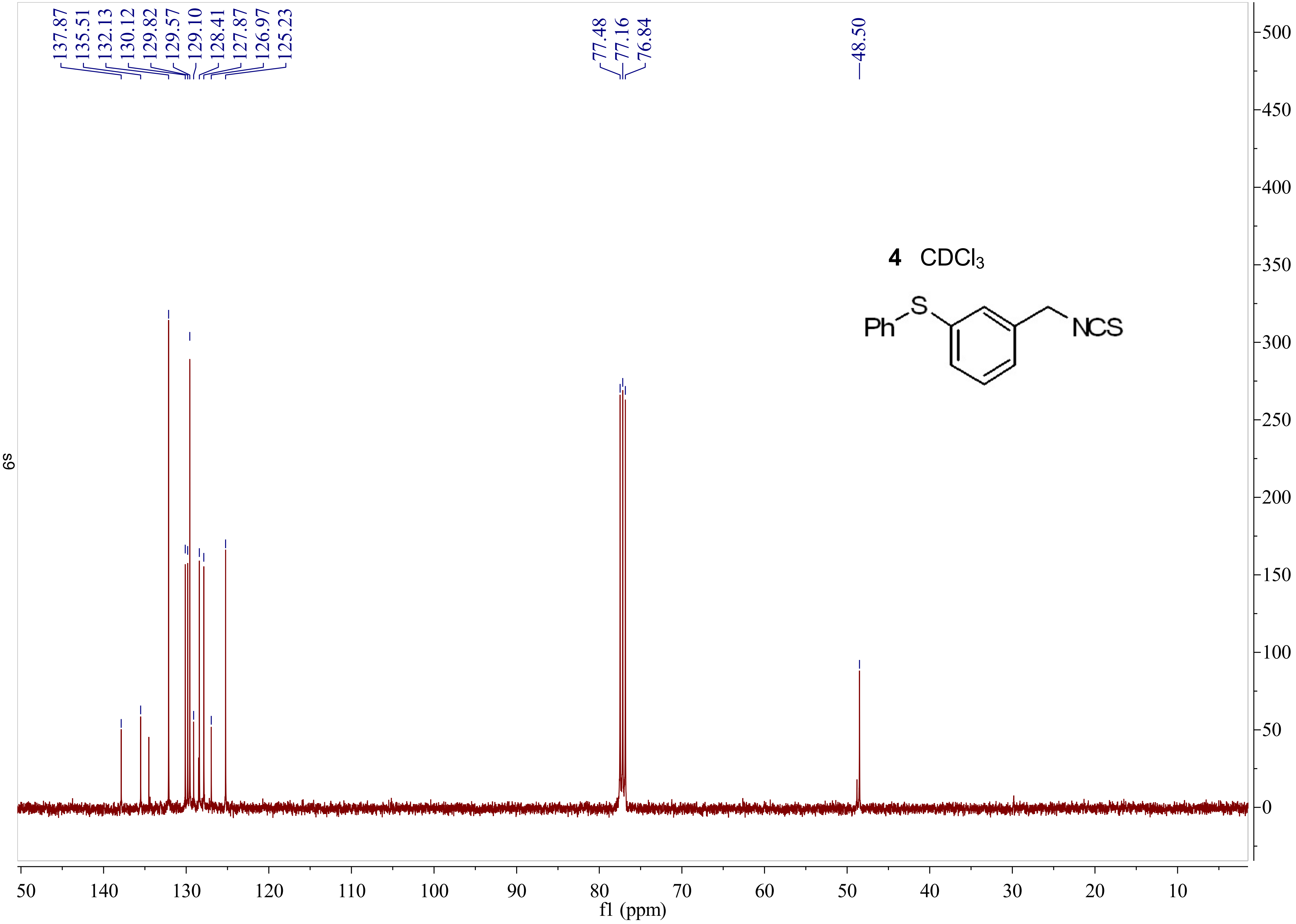
137.87
135.51
132.13
130.12
129.82
129.57
129.10
128.41
127.87
126.97
125.23
77.48
77.16
76.84
48.50
4 CDCl3
Ph
S
NCS
fl (ppm)

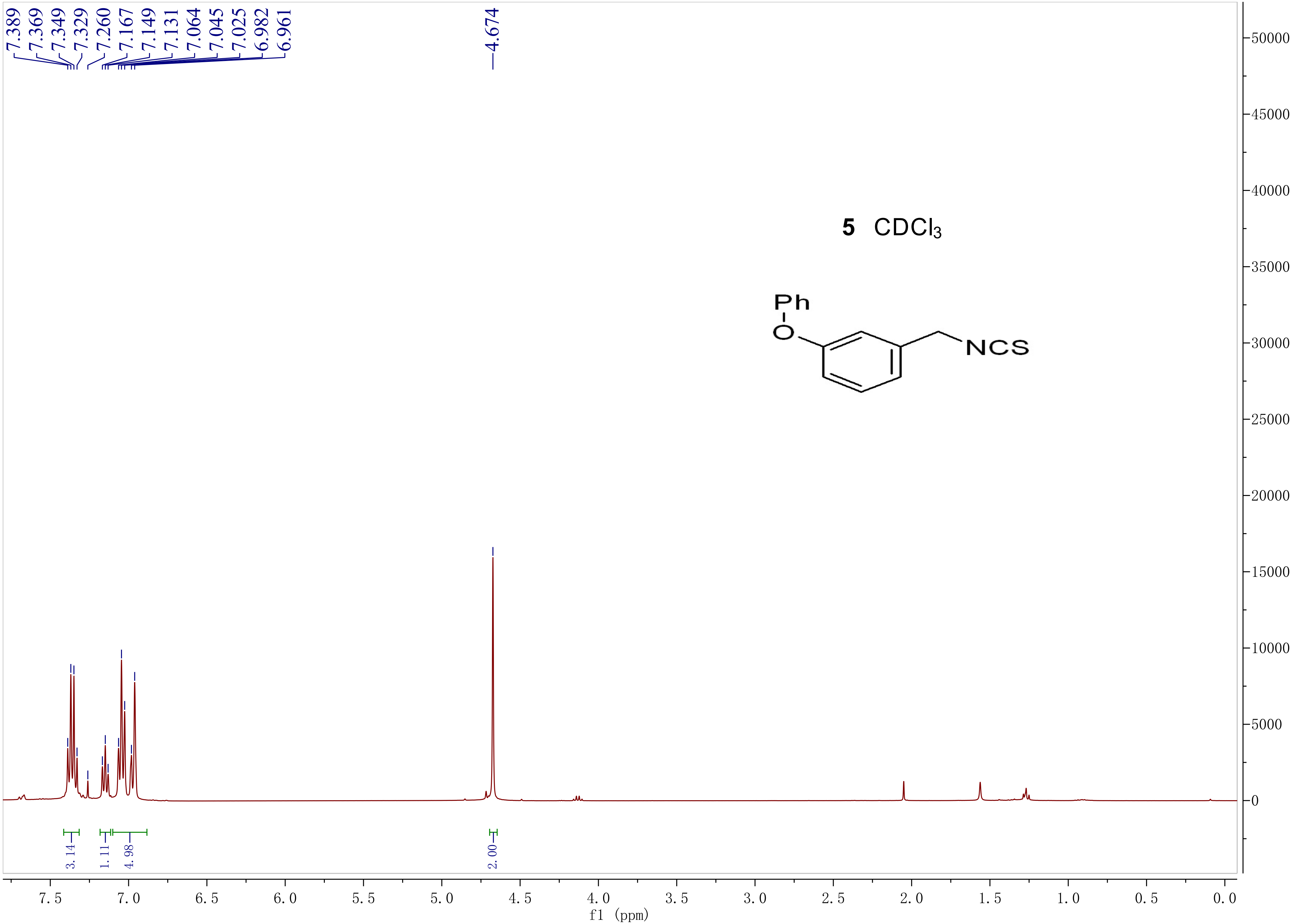
7.389
7.369
7.349
7.329
7.260
7.167
7.149
7.131
7.064
7.045
7.025
6.982
6.961
4.674
5 CDCl3
Ph
O
NCS
3.14
1.11
4.98
2.00
f1 (ppm)

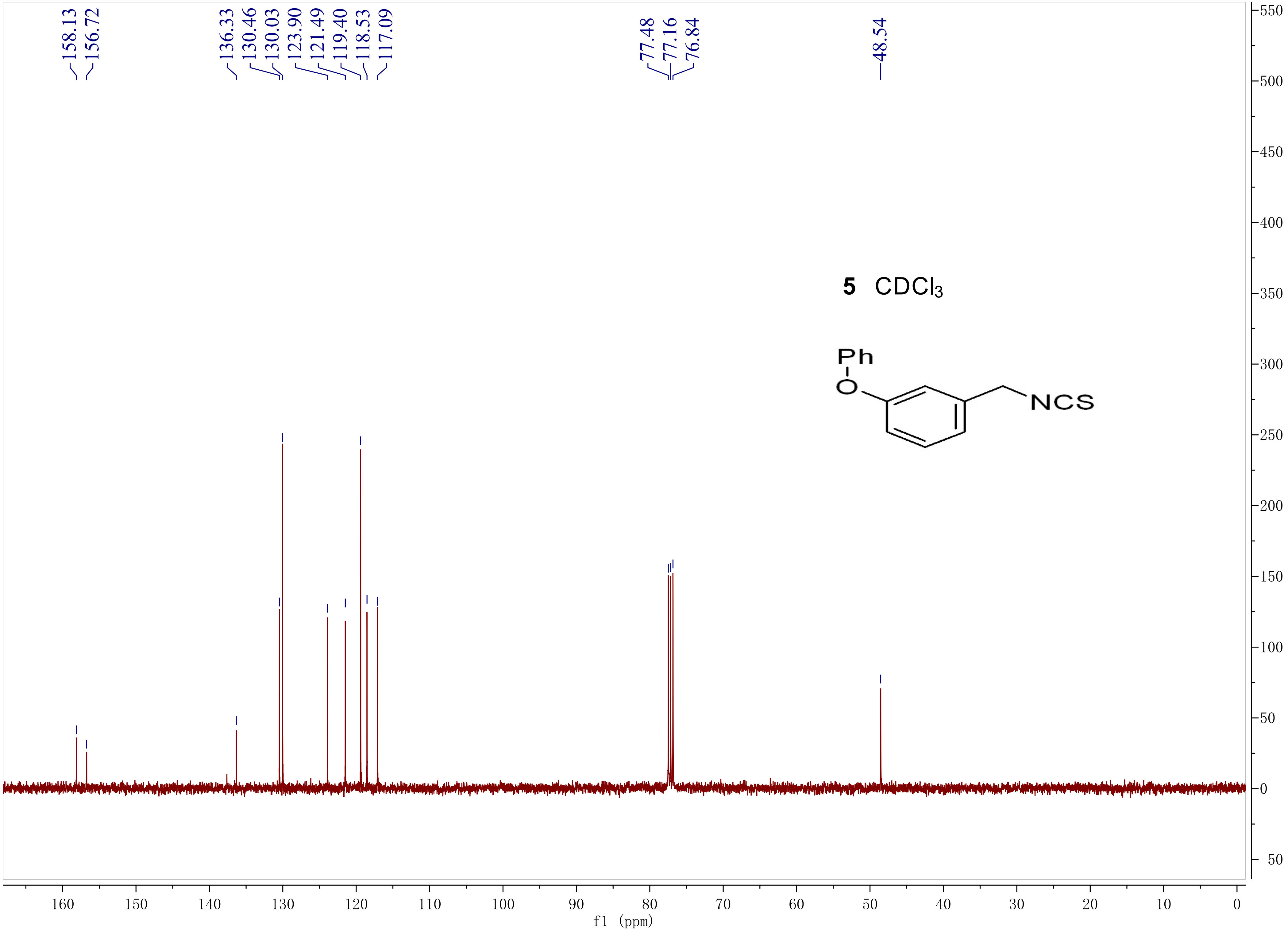
158.13
156.72
136.33
130.46
130.03
123.90
121.49
119.40
118.53
117.09
77.48
77.16
76.84
48.54
5 CDCl3
Ph
O
NCS
f1 (ppm)

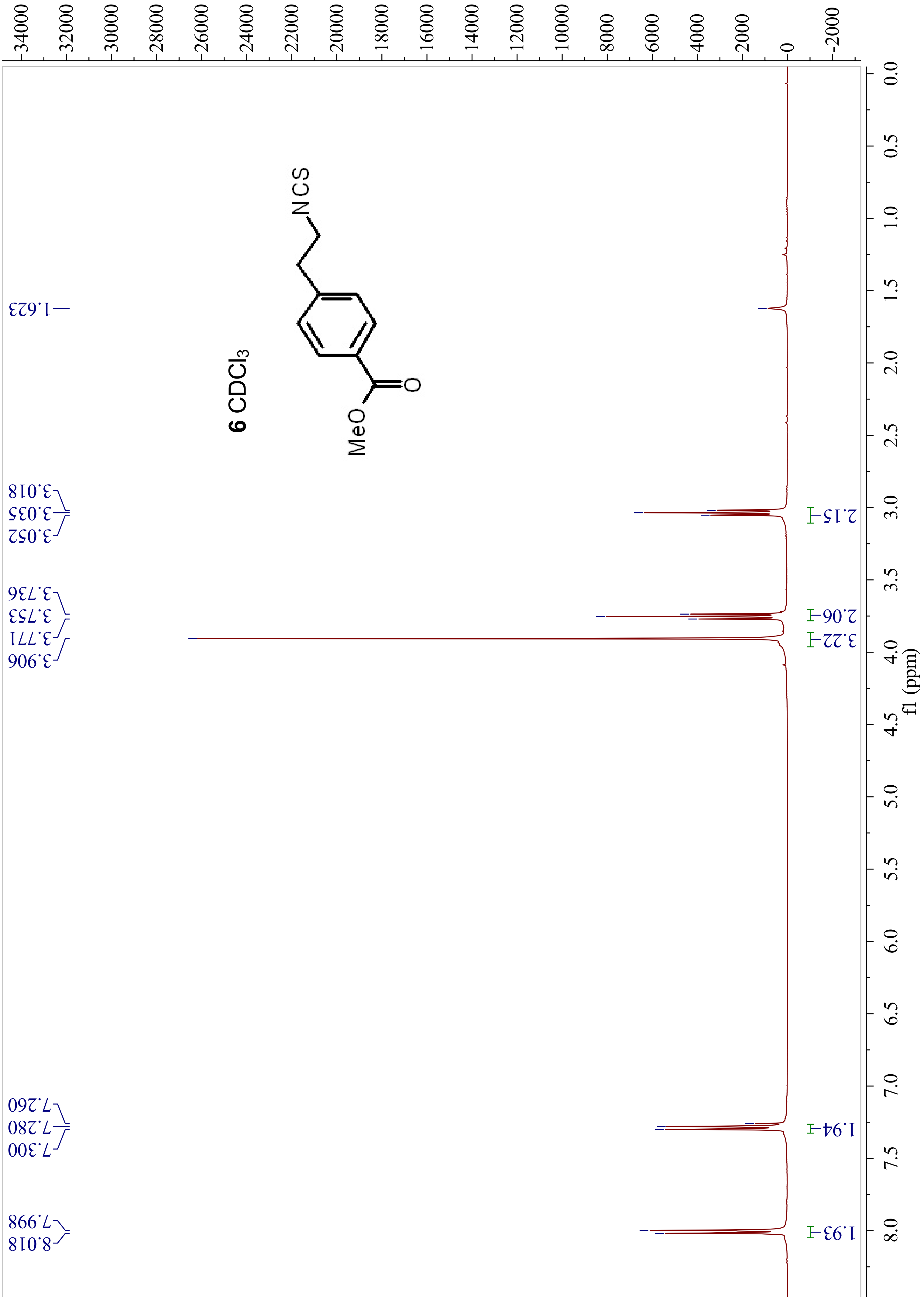
6 CDCl3
MeO
O
NCS
1.623
3.018
3.035
3.052
3.736
3.753
3.771
3.906
7.260
7.280
7.300
7.998
8.018
2.15
2.06
3.22
1.94
1.93
f1 (ppm)

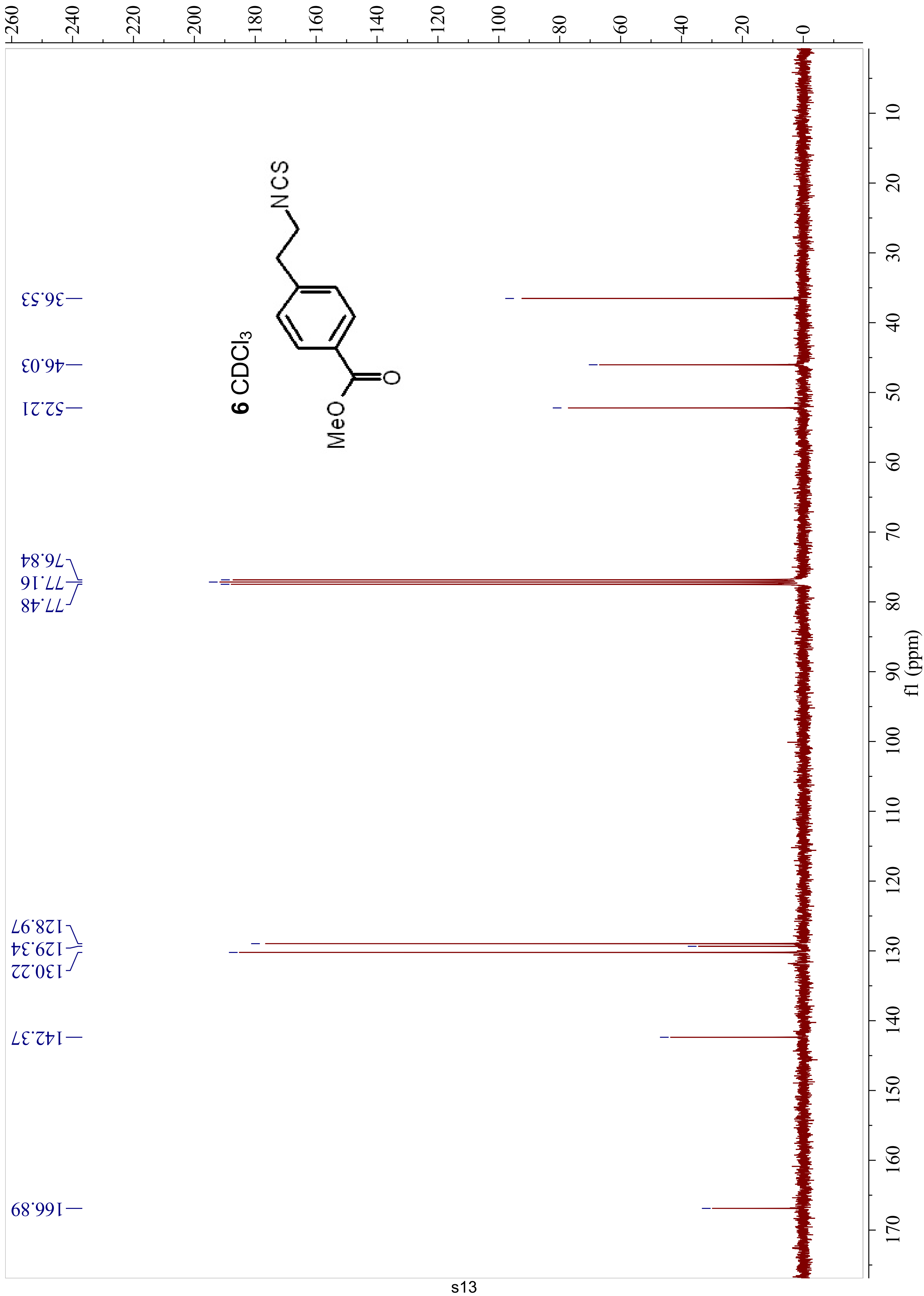
6 CDCl3
MeO
O
NCS
36.53
46.03
52.21
76.84
77.16
77.48
128.97
129.34
130.22
142.37
166.89
f1 (ppm)

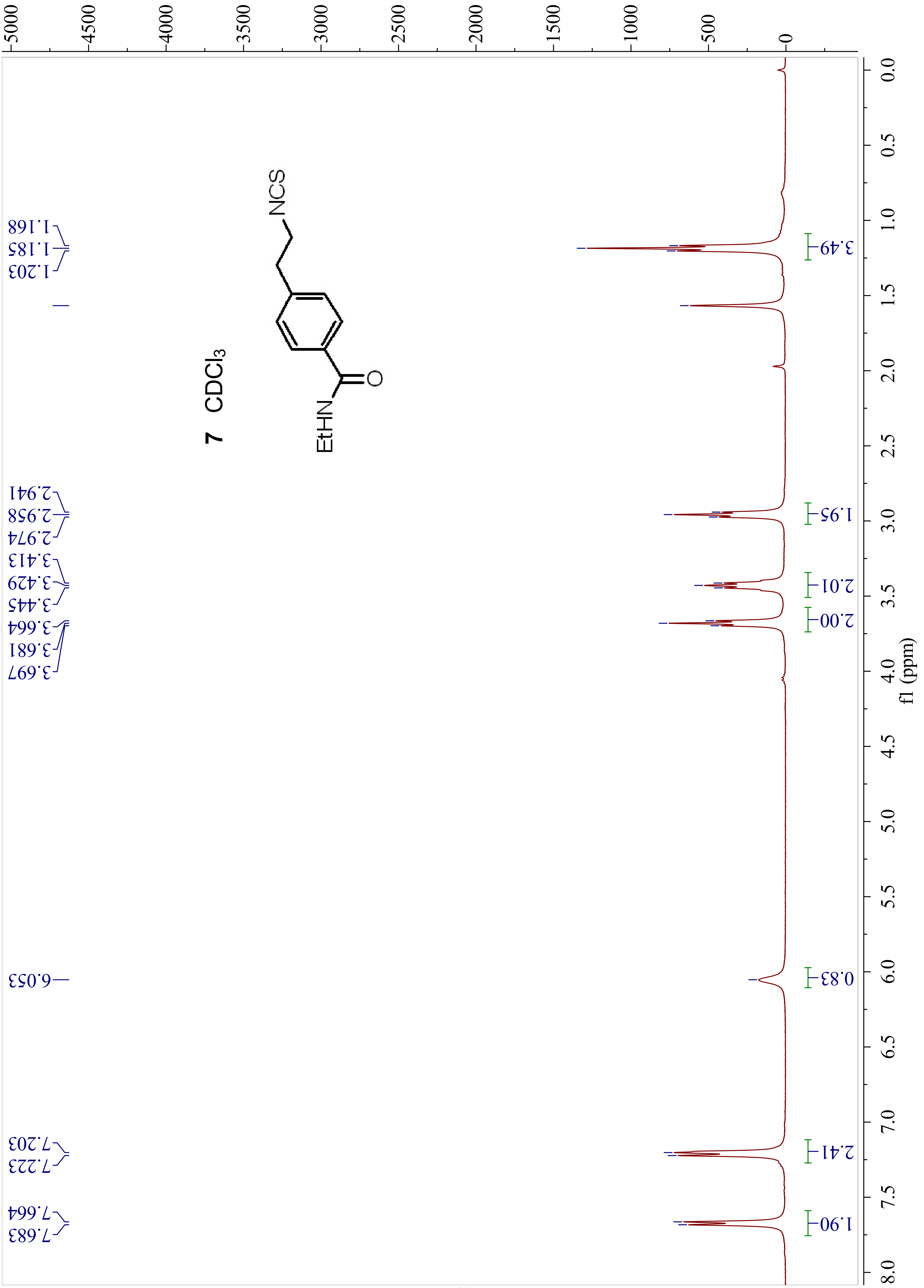
7 CDCl3
EtHN
O
NCS
1.168
1.185
1.203
2.941
2.958
2.974
3.413
3.429
3.445
3.664
3.681
3.697
6.053
7.203
7.223
7.664
7.683
3.49
1.95
2.01
2.00
0.83
2.41
1.90
f1 (ppm)

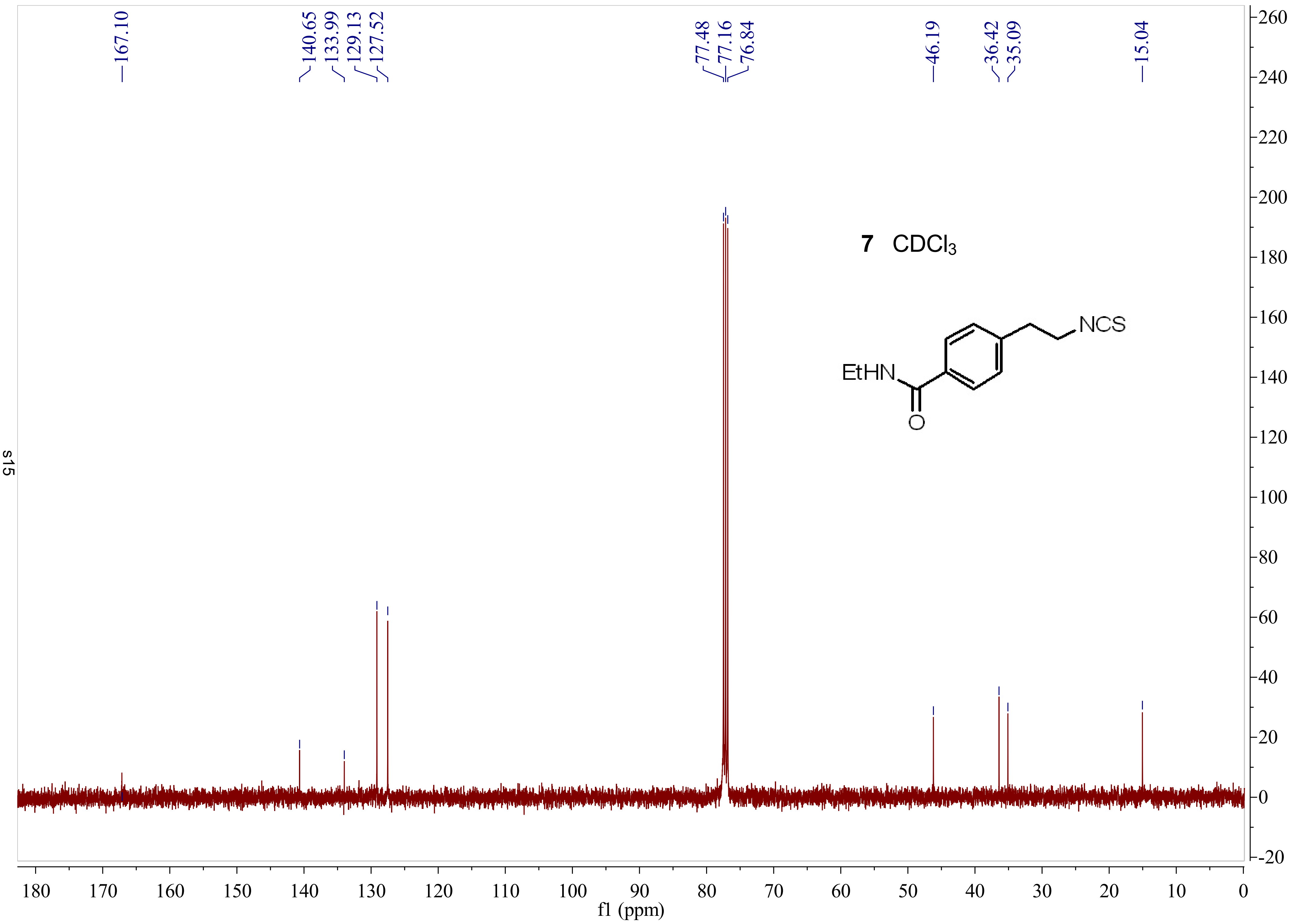
167.10
140.65
133.99
129.13
127.52
77.48
77.16
76.84
46.19
36.42
35.09
15.04
7 CDCl3
EtHN
NCS
O
f1 (ppm)

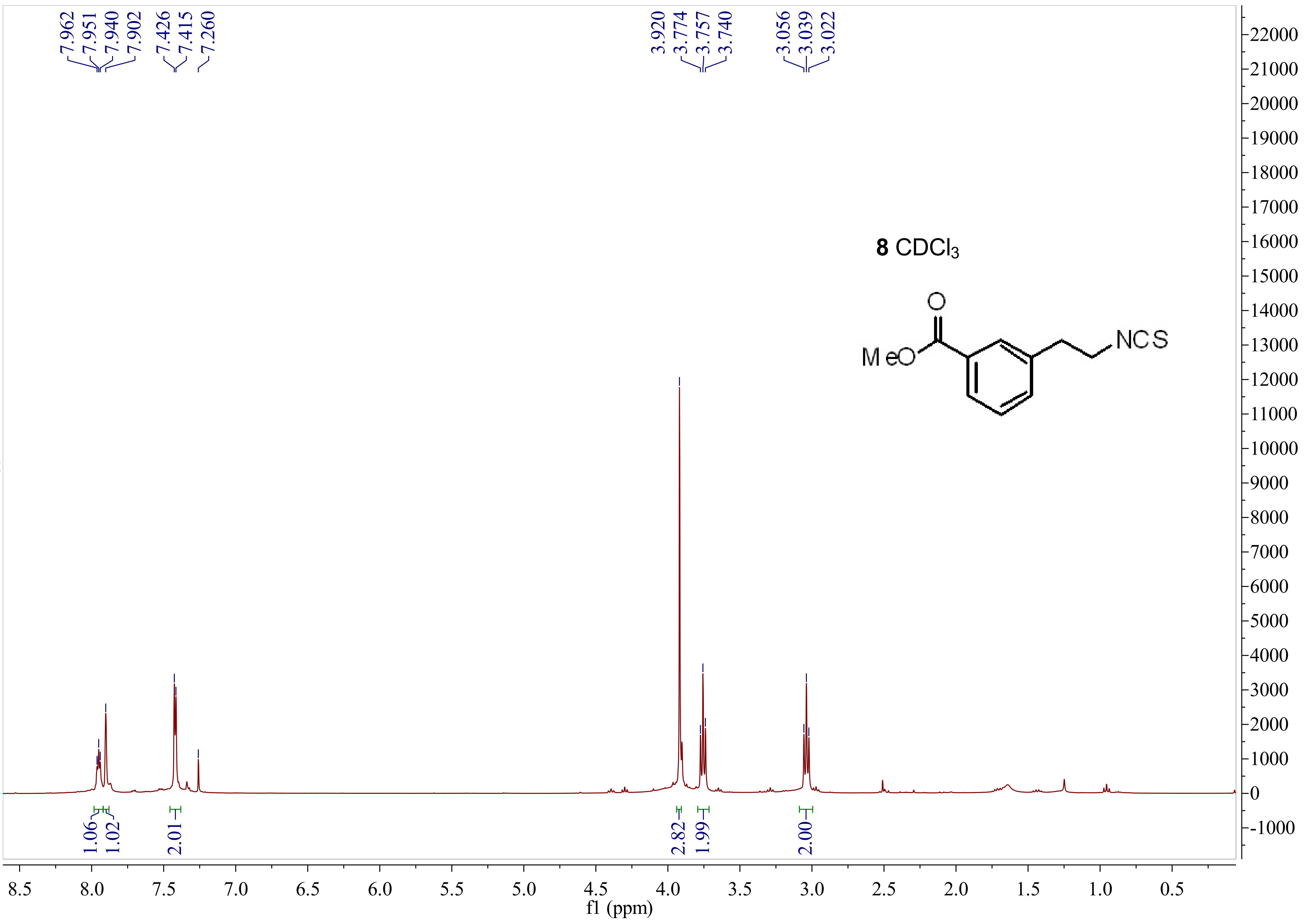
7.962
7.951
7.940
7.902
7.426
7.415
7.260
3.920
3.774
3.757
3.740
3.056
3.039
3.022
8 CDCl3
MeO
O
NCS
1.06
1.02
2.01
2.82
1.99
2.00
f1 (ppm)

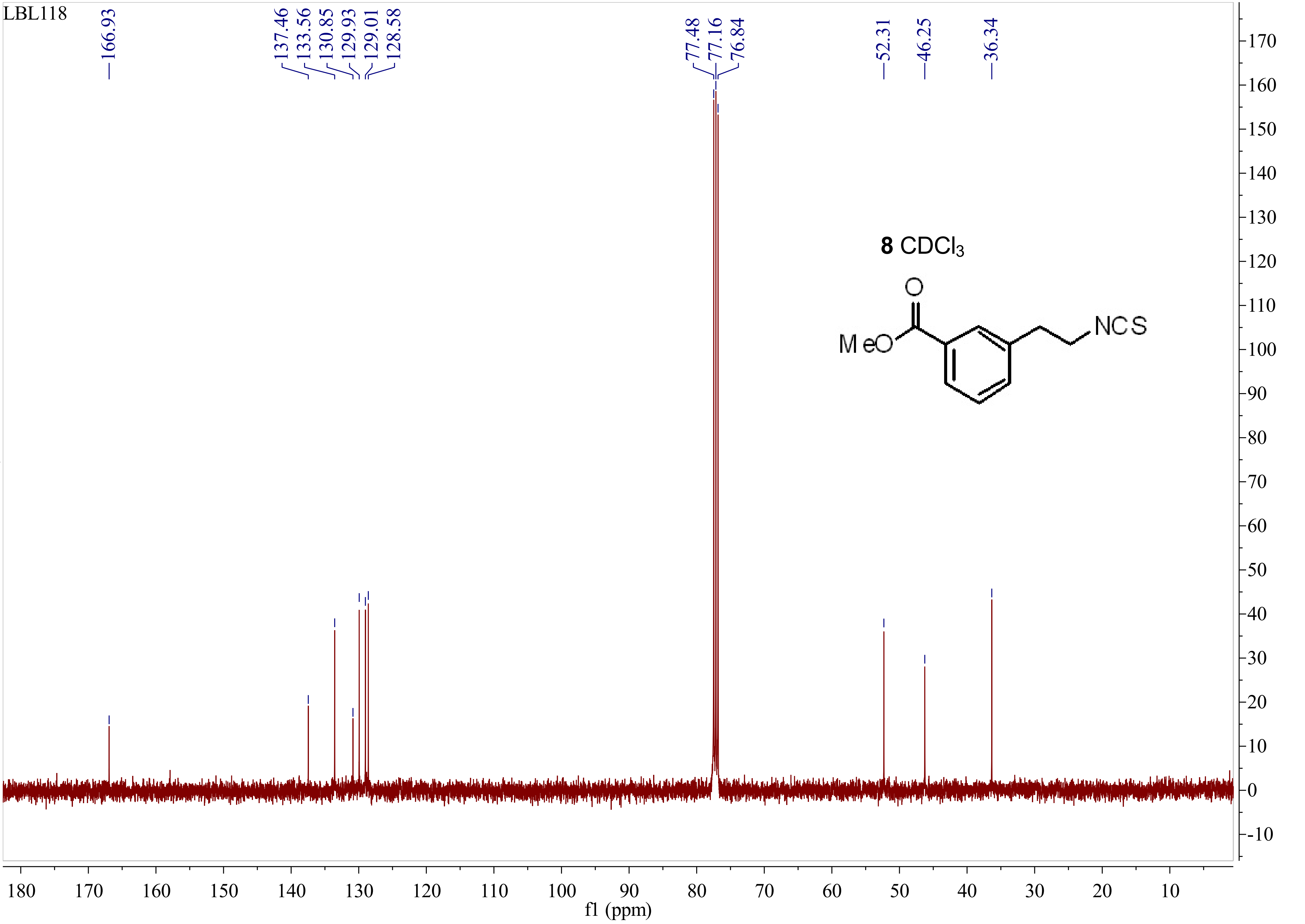
LBL118
166.93
137.46
133.56
130.85
129.93
129.01
128.58
77.48
77.16
76.84
52.31
46.25
36.34
8 CDCl3
MeO
O
NCS
f1 (ppm)

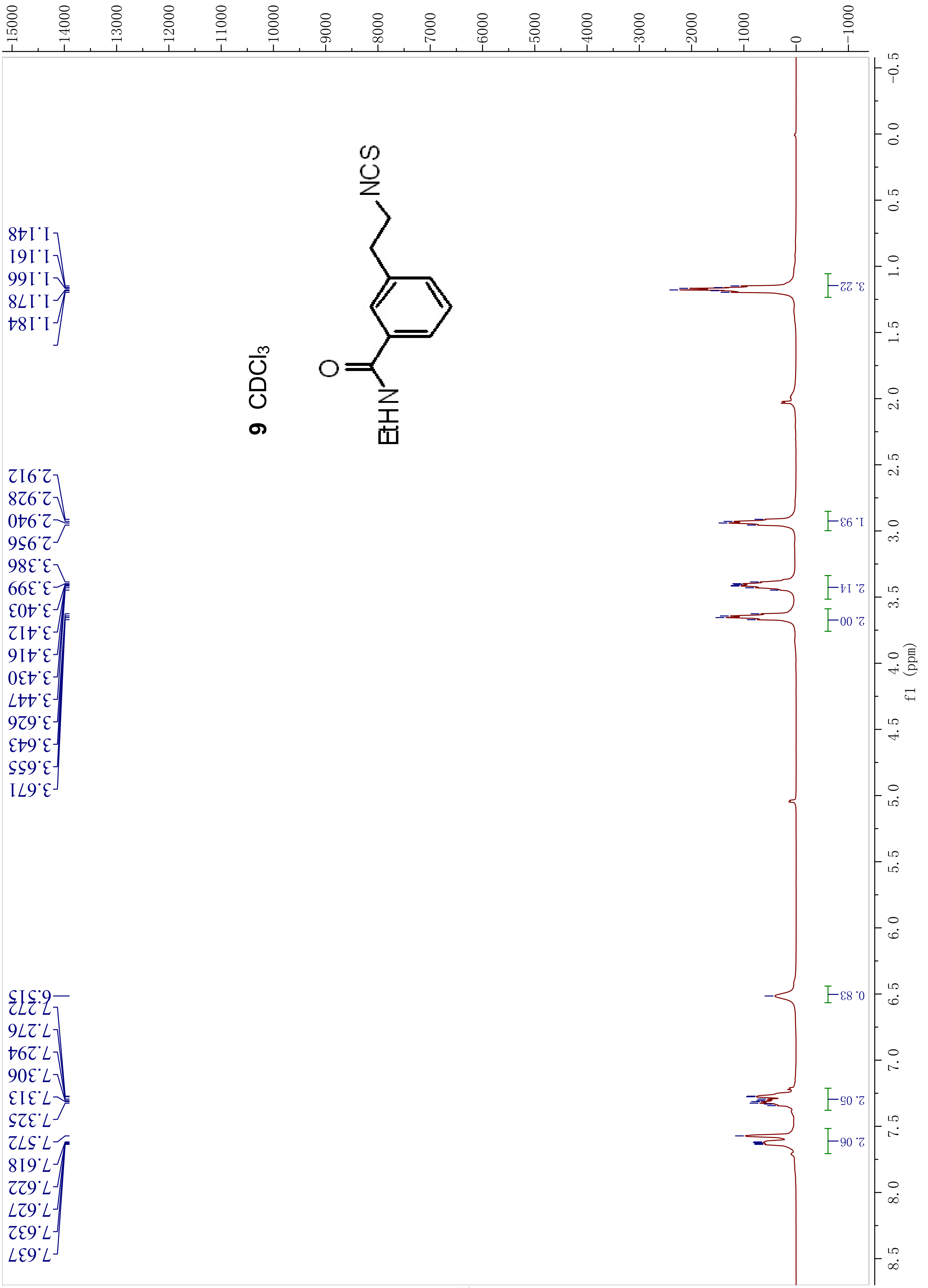
9 CDCl3
EtHN
O
NCS
1.148
1.161
1.166
1.178
1.184
2.912
2.928
2.940
2.956
3.386
3.399
3.403
3.412
3.416
3.430
3.447
3.626
3.643
3.655
3.671
6.515
7.272
7.276
7.294
7.306
7.313
7.325
7.572
7.618
7.622
7.627
7.632
7.637
3.22
1.93
2.14
2.00
0.83
2.05
2.06
f1 (ppm)

9 $CDCl_3$

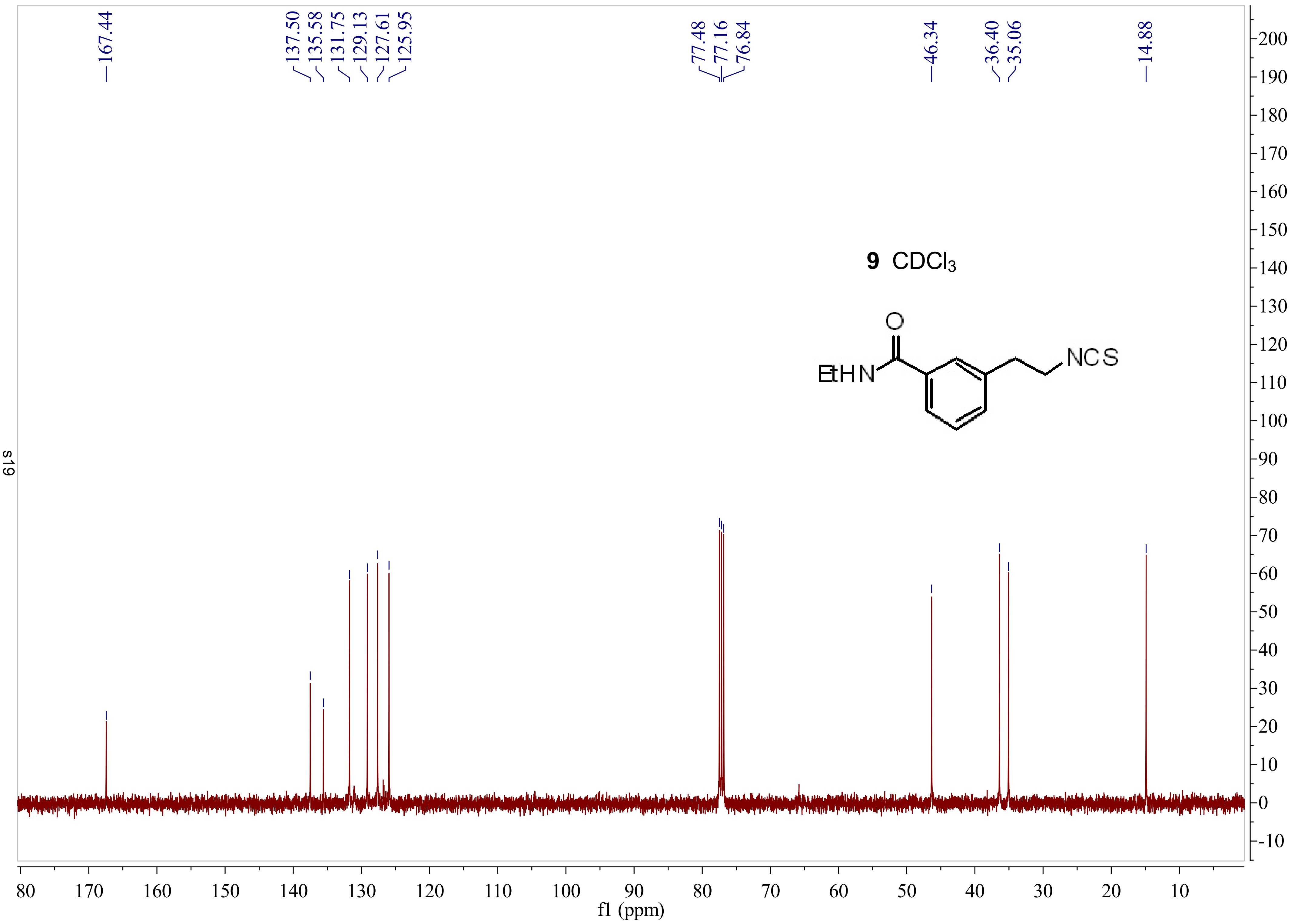

167.44
137.50
135.58
131.75
129.13
127.61
125.95
77.48
77.16
76.84
46.34
36.40
35.06
14.88
EtHN
O
NCS
f1 (ppm)

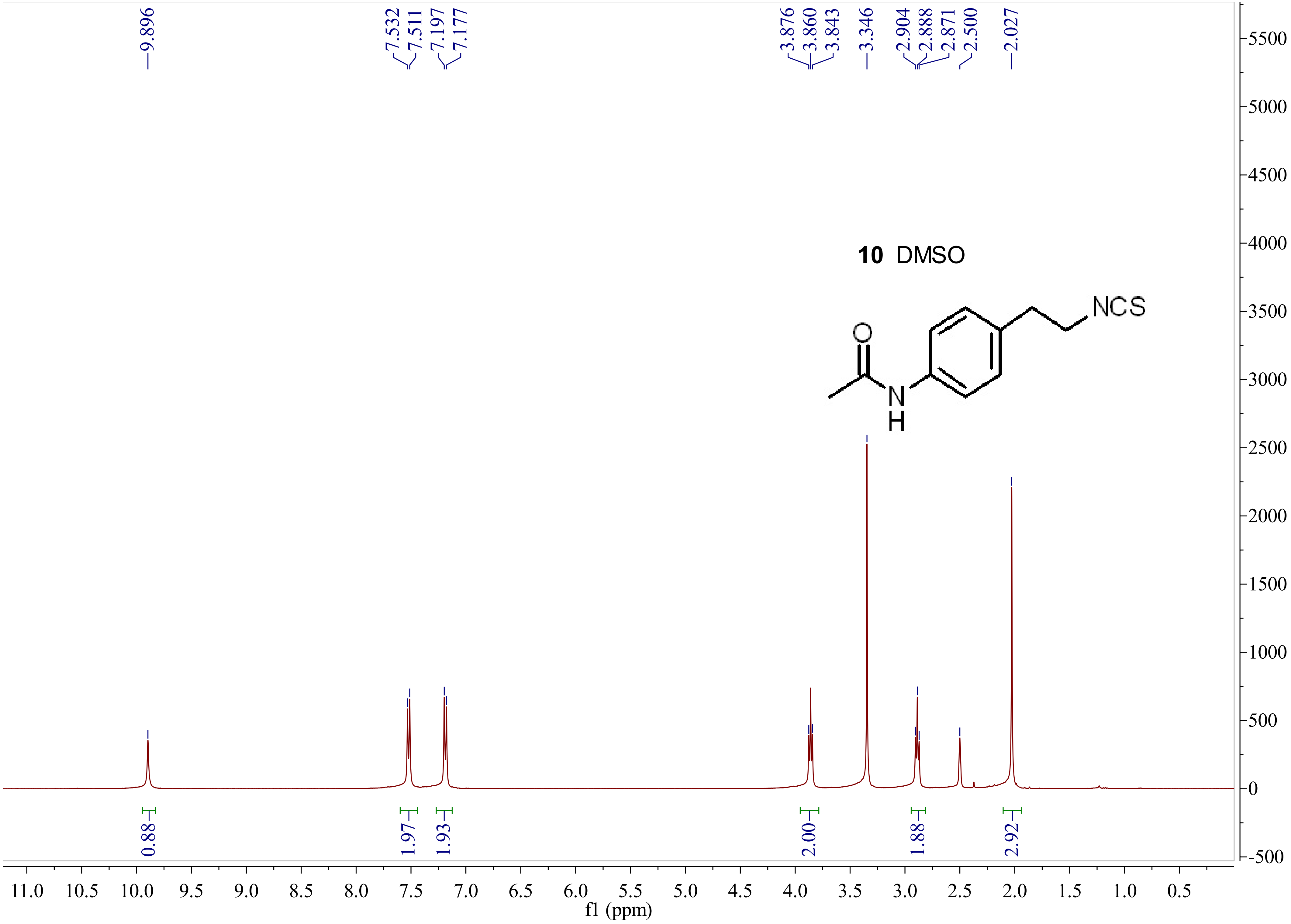
10 DMSO
NCS
9.896
7.532
7.511
7.197
7.177
3.876
3.860
3.843
3.346
2.904
2.888
2.871
2.500
2.027
0.88
1.97
1.93
2.00
1.88
2.92
f1 (ppm)

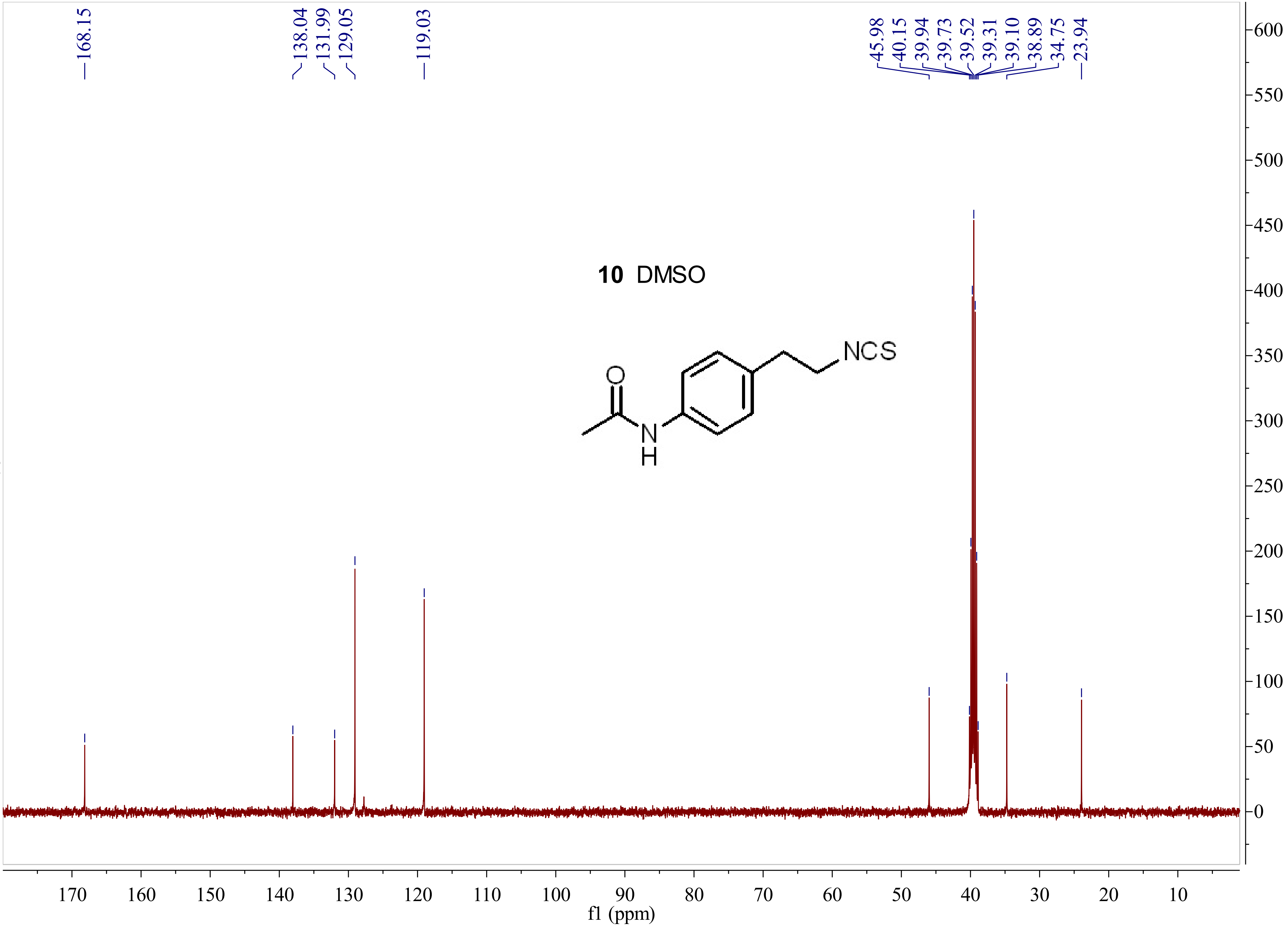
168.15
138.04
131.99
129.05
119.03
45.98
40.15
39.94
39.73
39.52
39.31
39.10
38.89
34.75
23.94
10 DMSO
NCS
O
N
H
f1 (ppm)

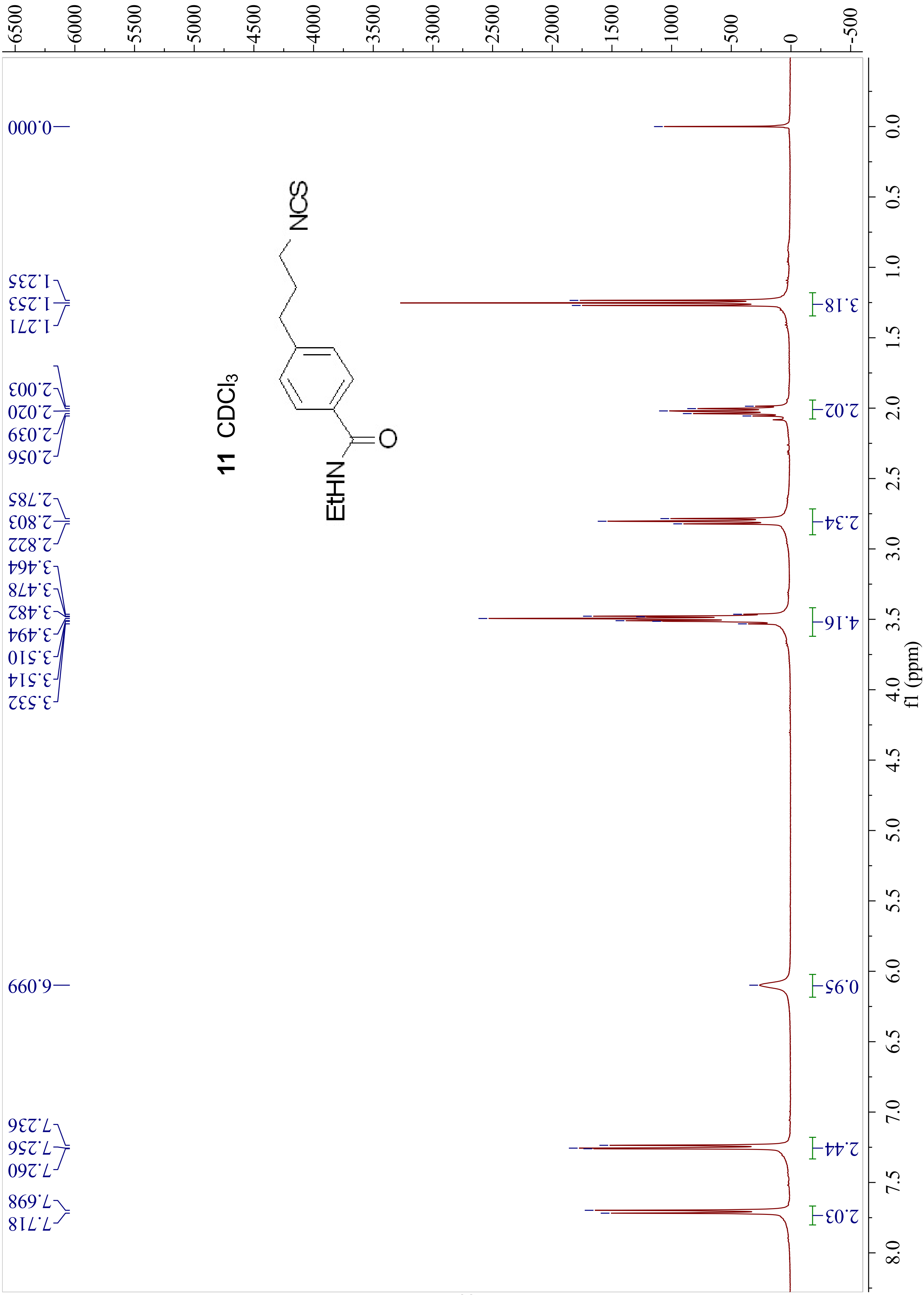

11 CDCl3
NCS
EtHN
O
0.000
1.235
1.253
1.271
2.003
2.020
2.039
2.056
2.785
2.803
2.822
3.464
3.478
3.482
3.494
3.510
3.514
3.532
6.099
7.236
7.256
7.260
7.698
7.718
3.18
2.02
2.34
4.16
0.95
2.44
2.03
f1 (ppm)

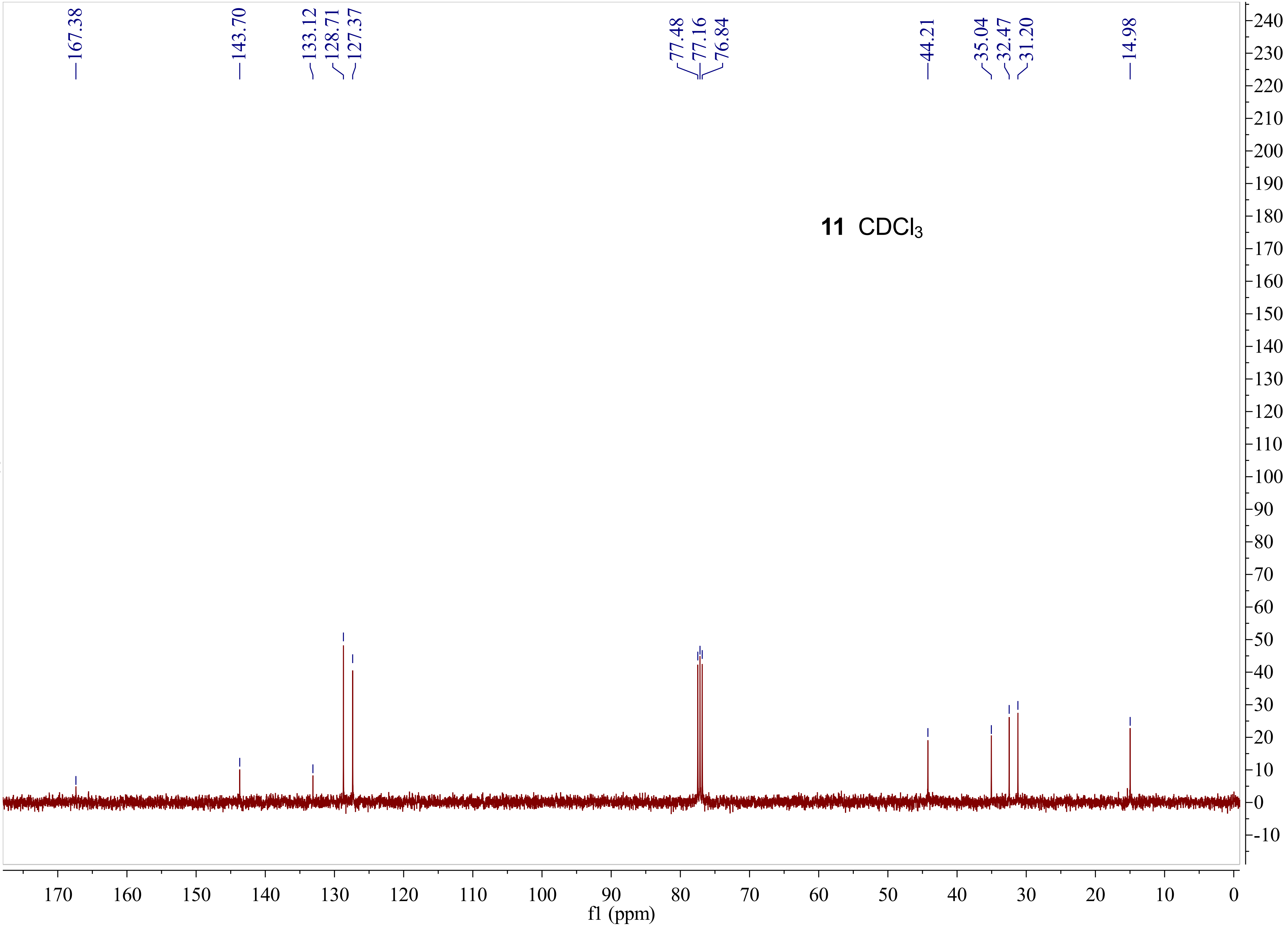
167.38
143.70
133.12
128.71
127.37
77.48
77.16
76.84
44.21
35.04
32.47
31.20
14.98
11 CDCl3
f1 (ppm)

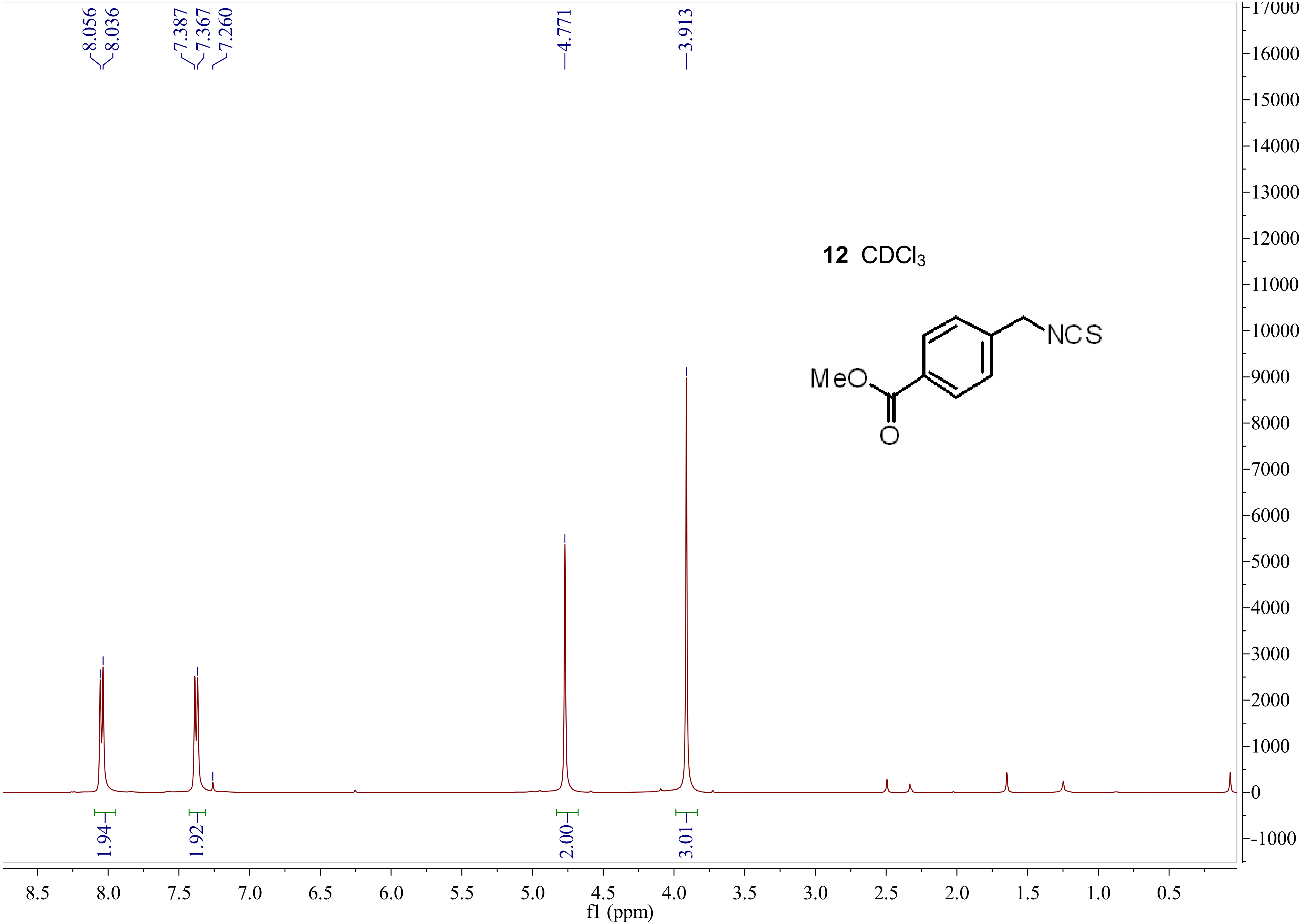
8.056
8.036
7.387
7.367
7.260
4.771
3.913
12 CDCl3
MeO
O
NCS
1.94
1.92
2.00
3.01
f1 (ppm)

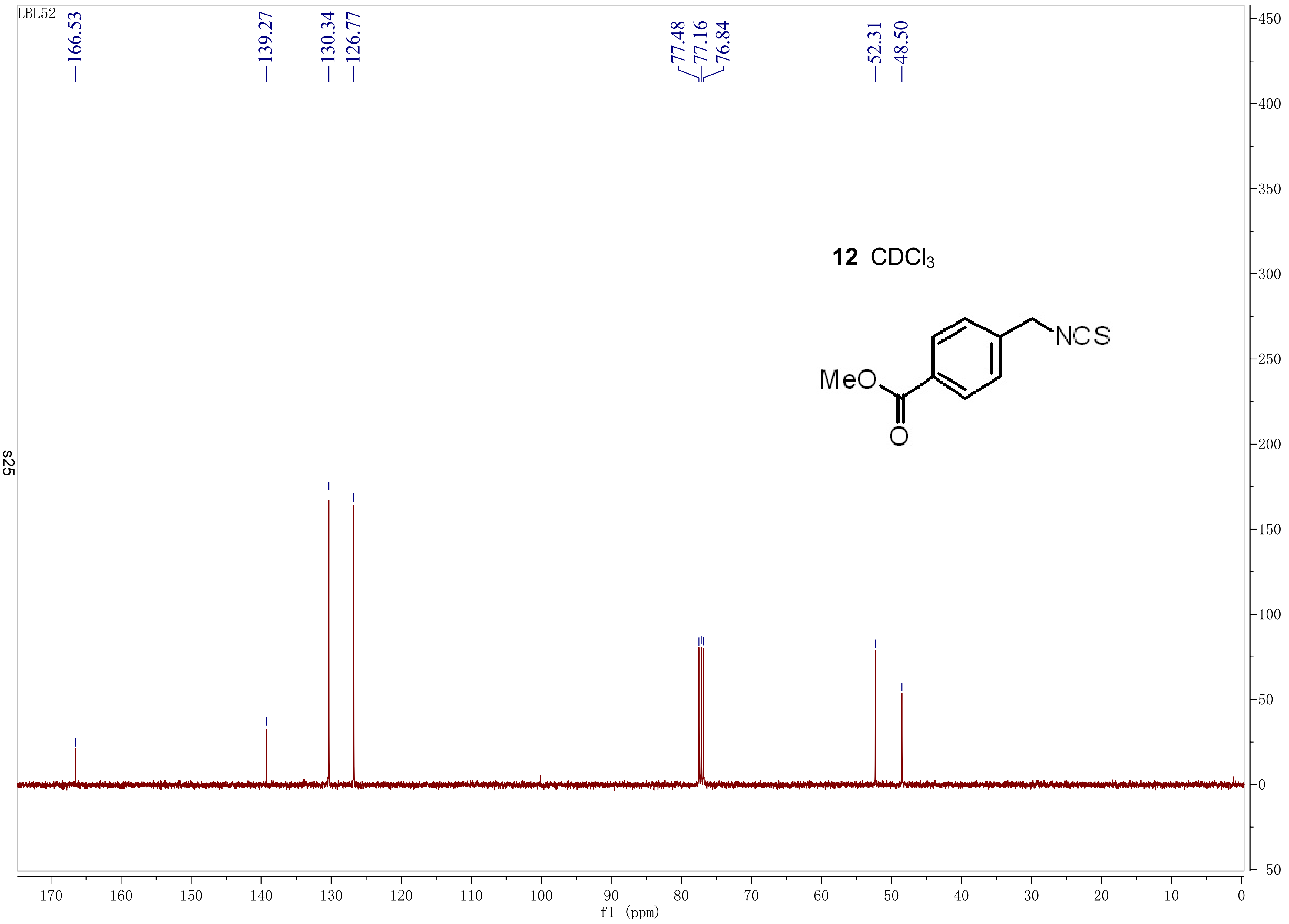
LBL52
166.53
139.27
130.34
126.77
77.48
77.16
76.84
52.31
48.50
12 CDCl3
MeO
O
NCS
f1 (ppm)

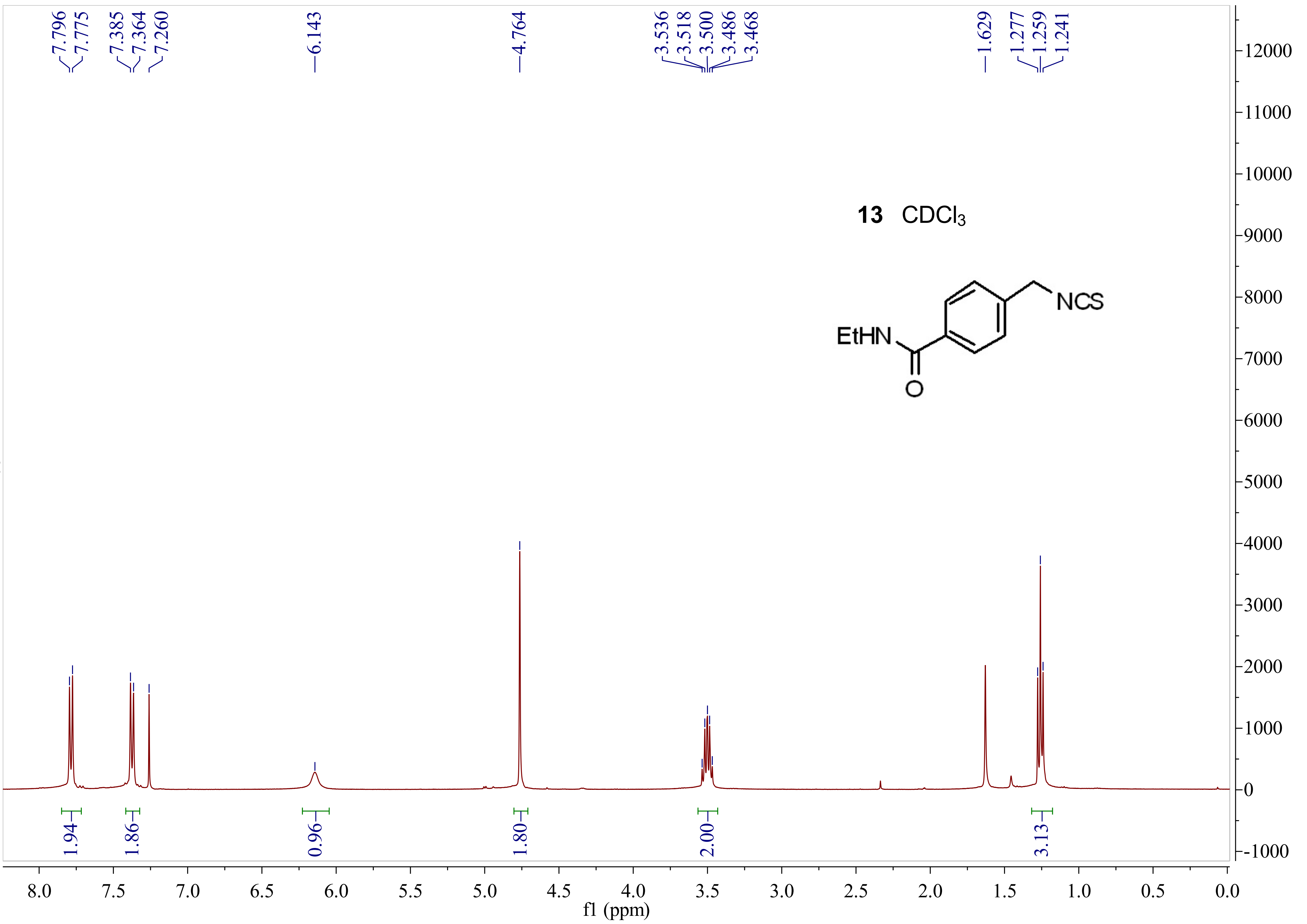
13 CDCl3
EtHN
NCS
O
7.796
7.775
7.385
7.364
7.260
6.143
4.764
3.536
3.518
3.500
3.486
3.468
1.629
1.277
1.259
1.241
1.94
1.86
0.96
1.80
2.00
3.13
f1 (ppm)

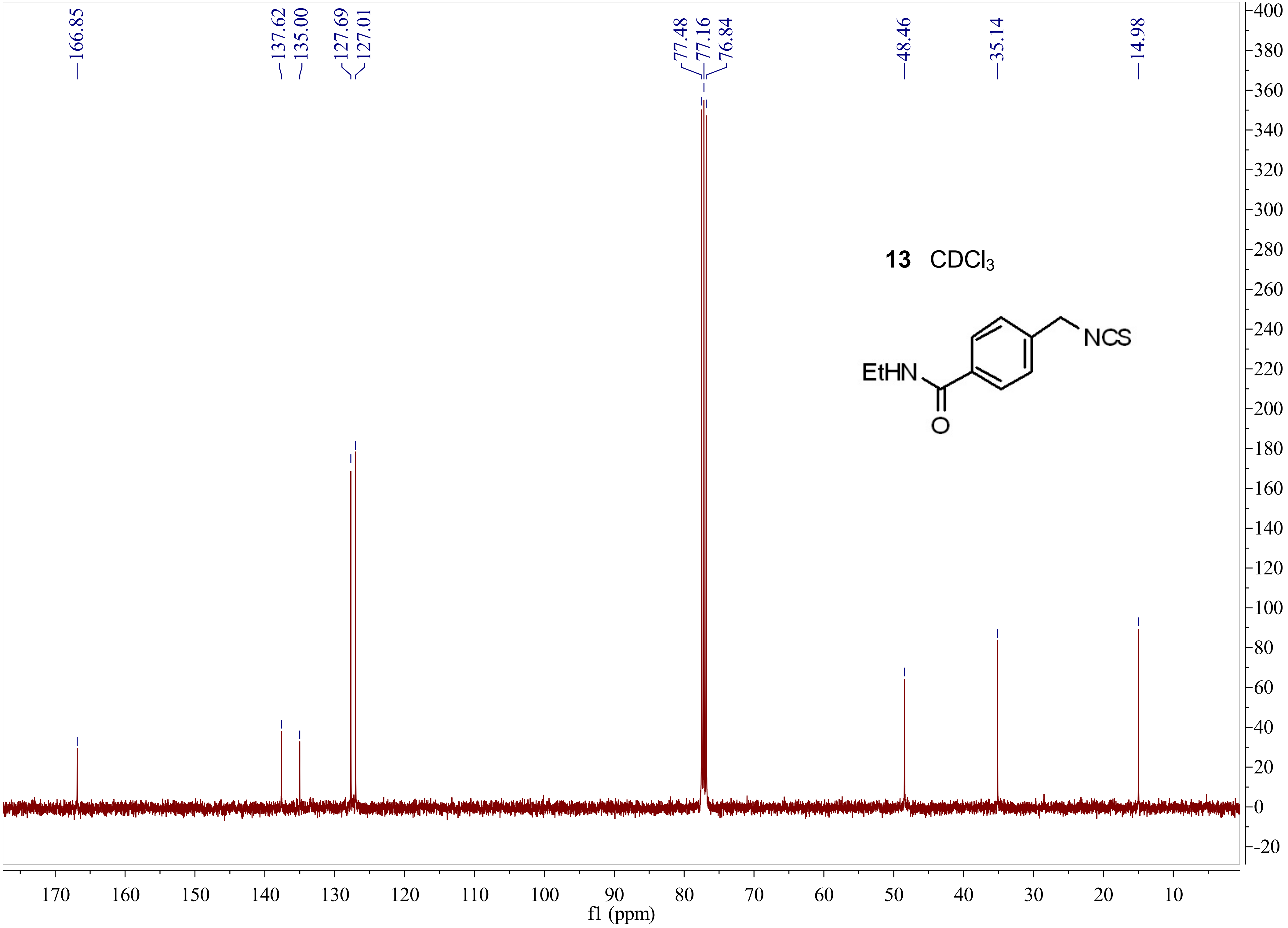
166.85
137.62
135.00
127.69
127.01
77.48
77.16
76.84
48.46
35.14
14.98
13 CDCl3
EtHN
O
NCS
f1 (ppm)

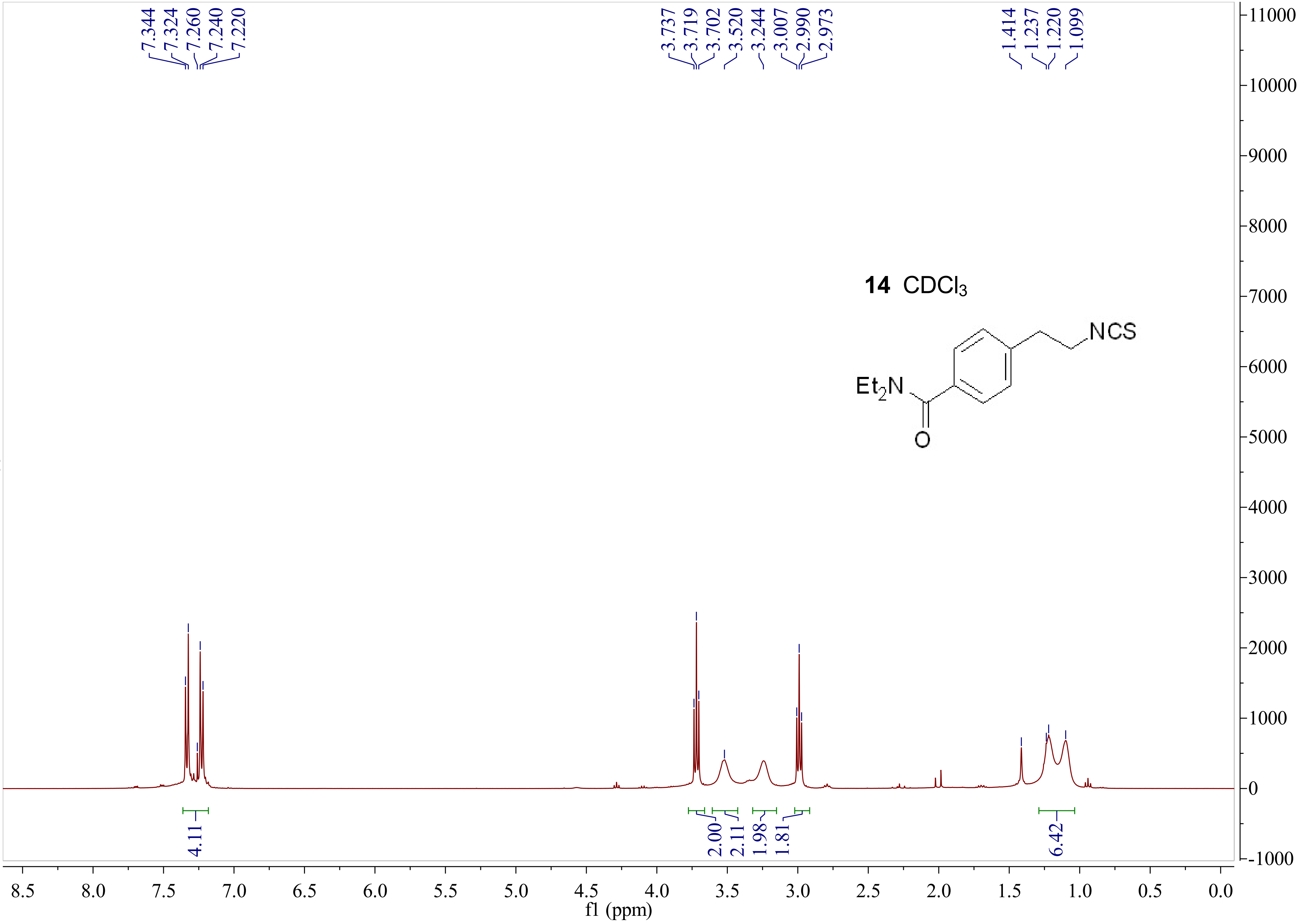
7.344
7.324
7.260
7.240
7.220
3.737
3.719
3.702
3.520
3.244
3.007
2.990
2.973
1.414
1.237
1.220
1.099
14 CDCl3
NCS
Et2N
O
4.11
2.00
2.11
1.98
1.81
6.42
fl (ppm)

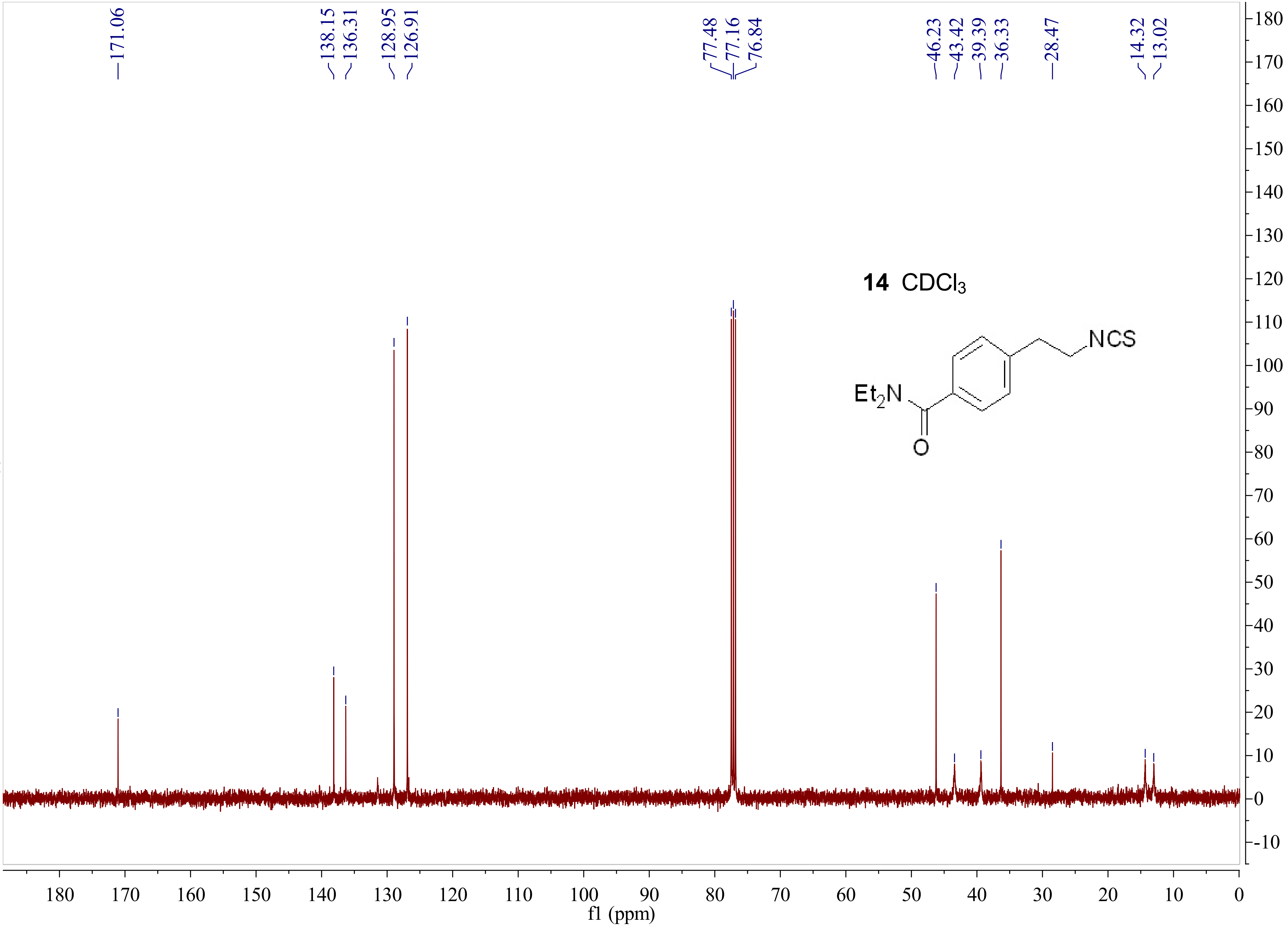
171.06
138.15
136.31
128.95
126.91
77.48
77.16
76.84
46.23
43.42
39.39
36.33
28.47
14.32
13.02
14 CDCl3
Et2N
NCS
O
f1 (ppm)

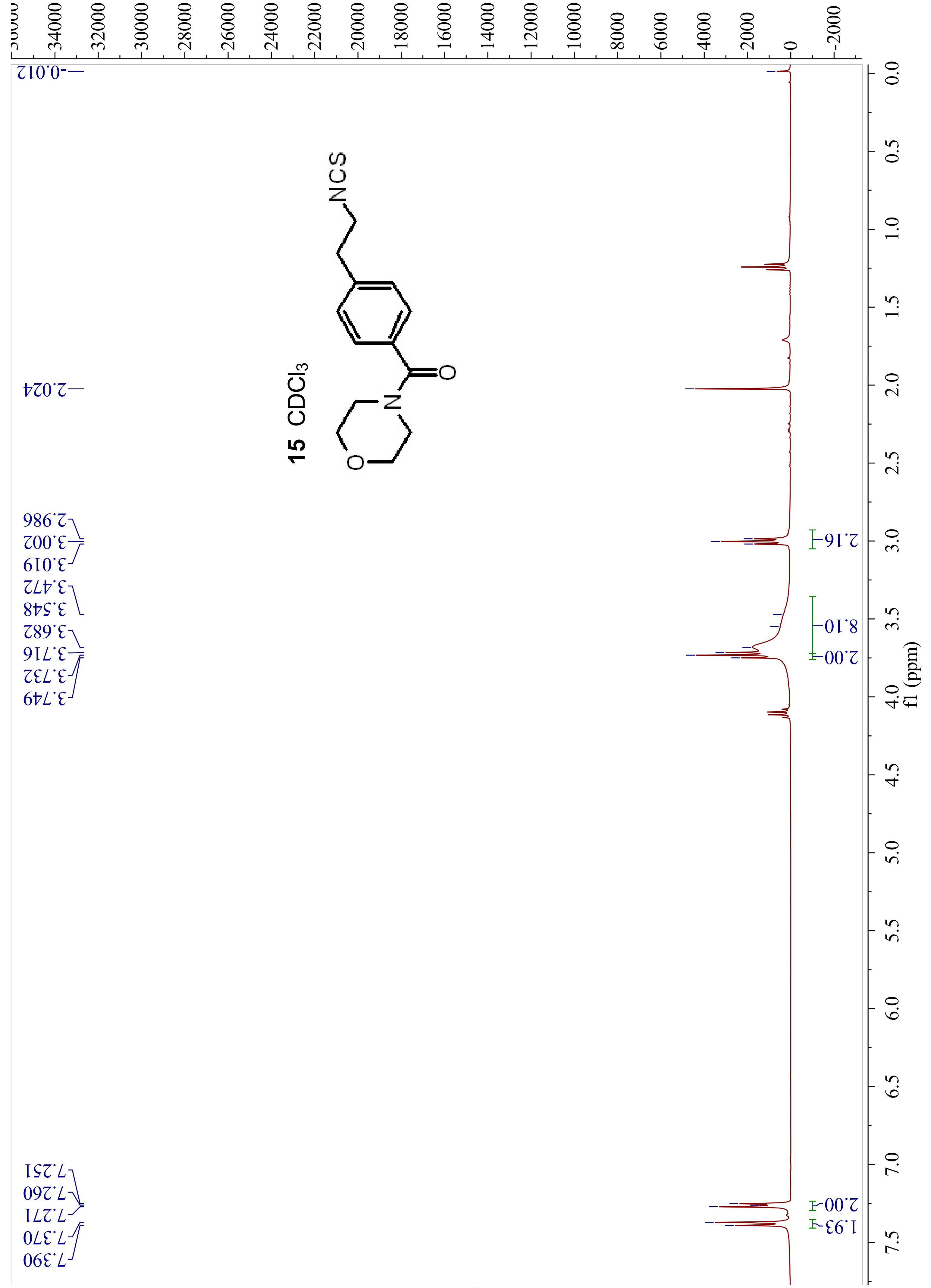
15 CDCl3
NCS
O
N
O
-0.012
2.024
2.986
3.002
3.019
3.472
3.548
3.682
3.716
3.732
3.749
7.251
7.260
7.271
7.370
7.390
2.16
8.10
2.00
2.00
1.93
fl (ppm)

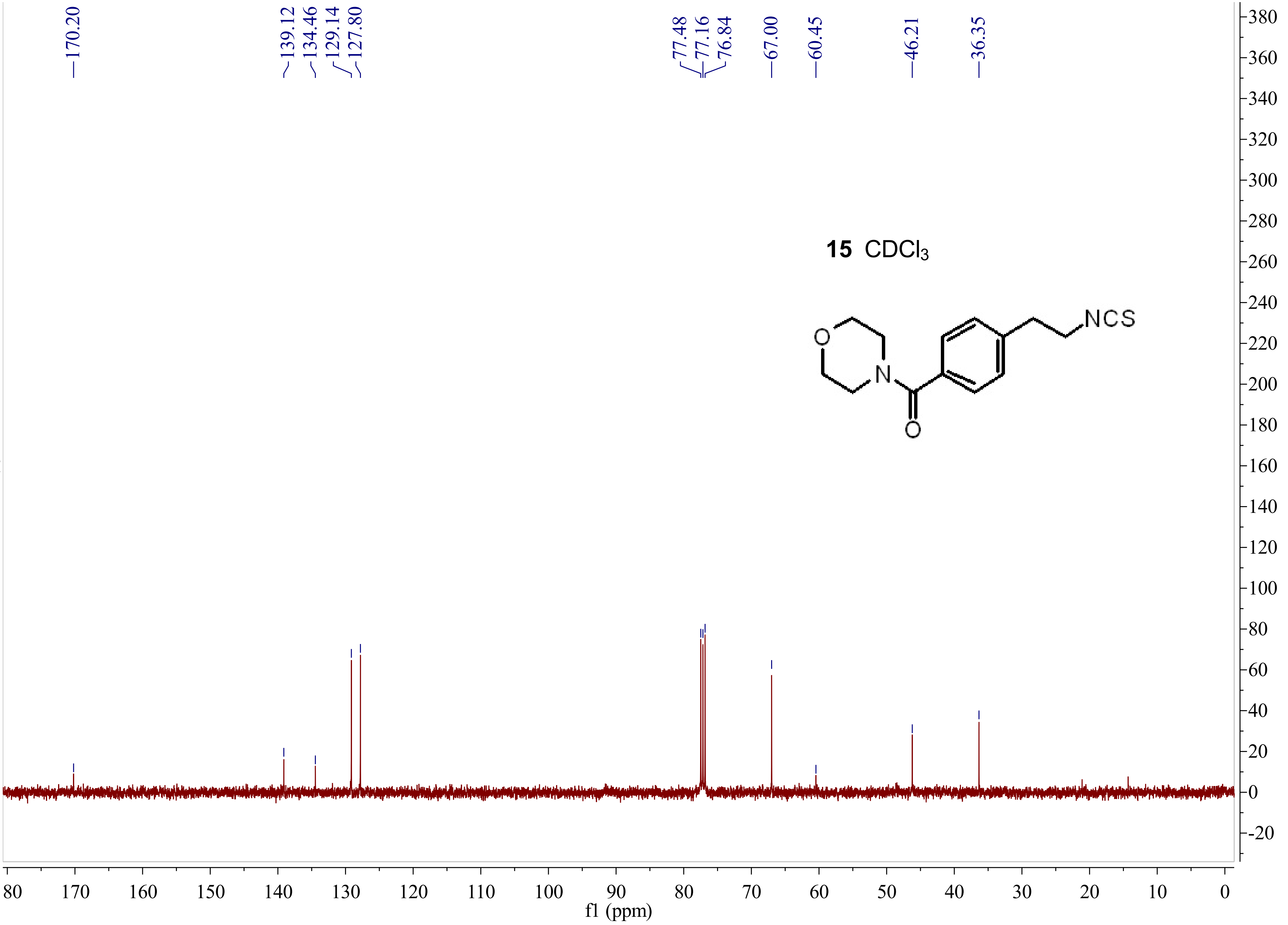
170.20
139.12
134.46
129.14
127.80
77.48
77.16
76.84
67.00
60.45
46.21
36.35
15 CDCl3
NCS
O
N
O
f1 (ppm)

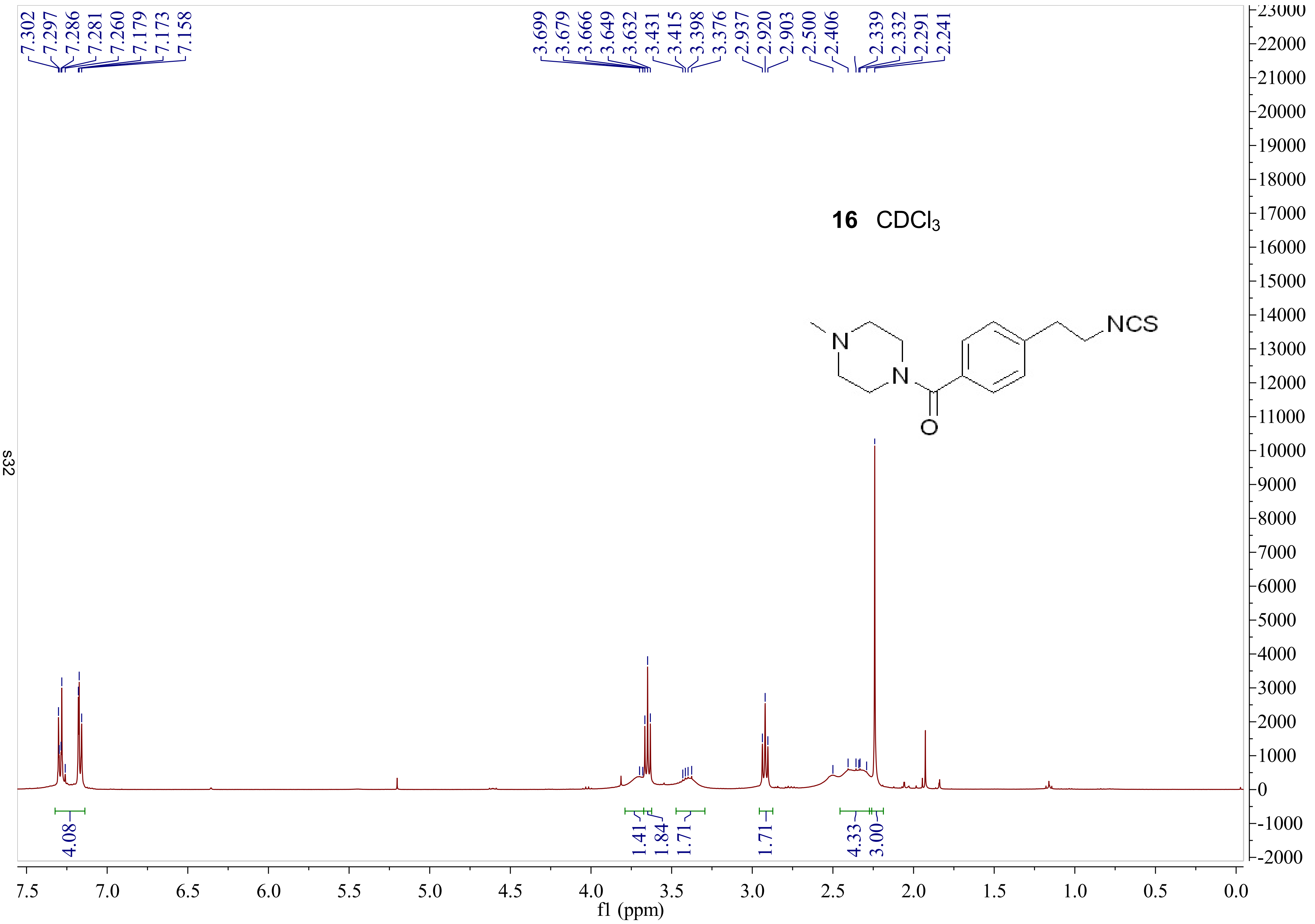
7.302
7.297
7.286
7.281
7.260
7.179
7.173
7.158
3.699
3.679
3.666
3.649
3.632
3.431
3.415
3.398
3.376
2.937
2.920
2.903
2.500
2.406
2.339
2.332
2.291
2.241
16 CDCl3
NCS
N
N
O
4.08
1.41
1.84
1.71
1.71
4.33
3.00
f1 (ppm)

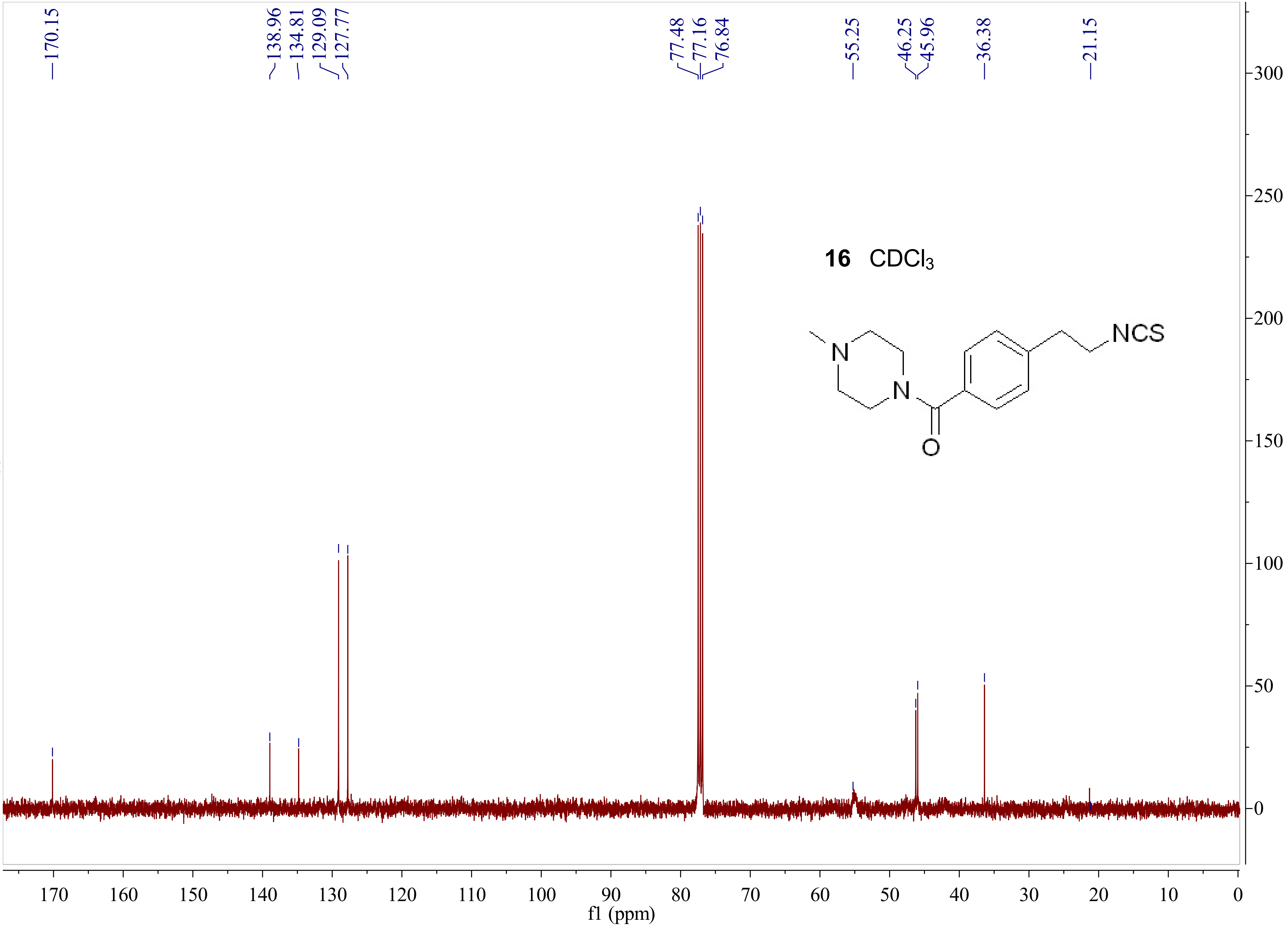
170.15
138.96
134.81
129.09
127.77
77.48
77.16
76.84
55.25
46.25
45.96
36.38
21.15
16 CDCl3
NCS
N
N
O
f1 (ppm)

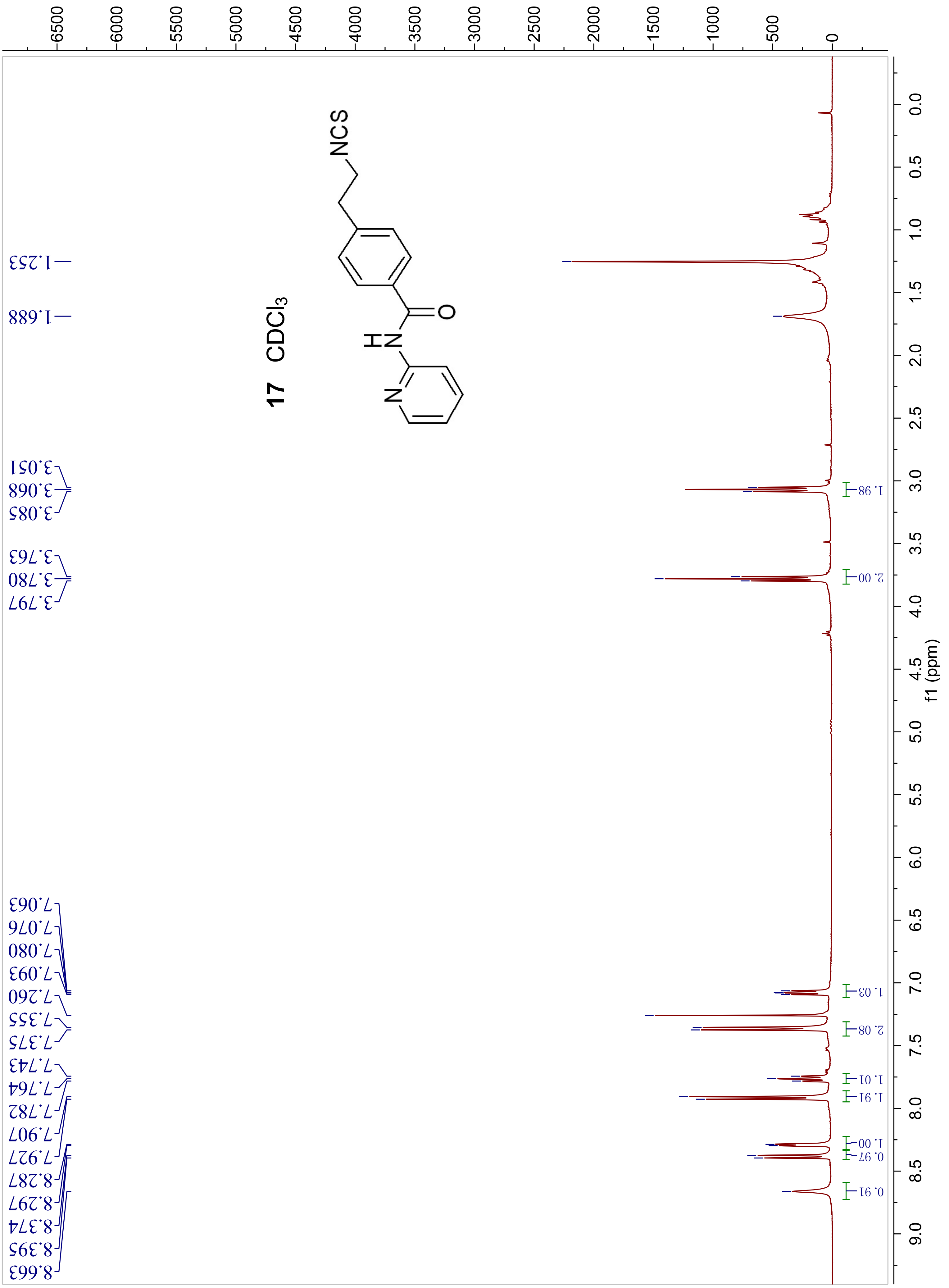
17 CDCl3
NCS
N
H
N
O
1.253
1.688
3.051
3.068
3.085
3.763
3.780
3.797
7.063
7.076
7.080
7.093
7.260
7.355
7.375
7.743
7.764
7.782
7.907
7.927
8.287
8.297
8.374
8.395
8.663
1.98
2.00
1.03
2.08
1.01
1.91
1.00
0.97
0.91
f1 (ppm)

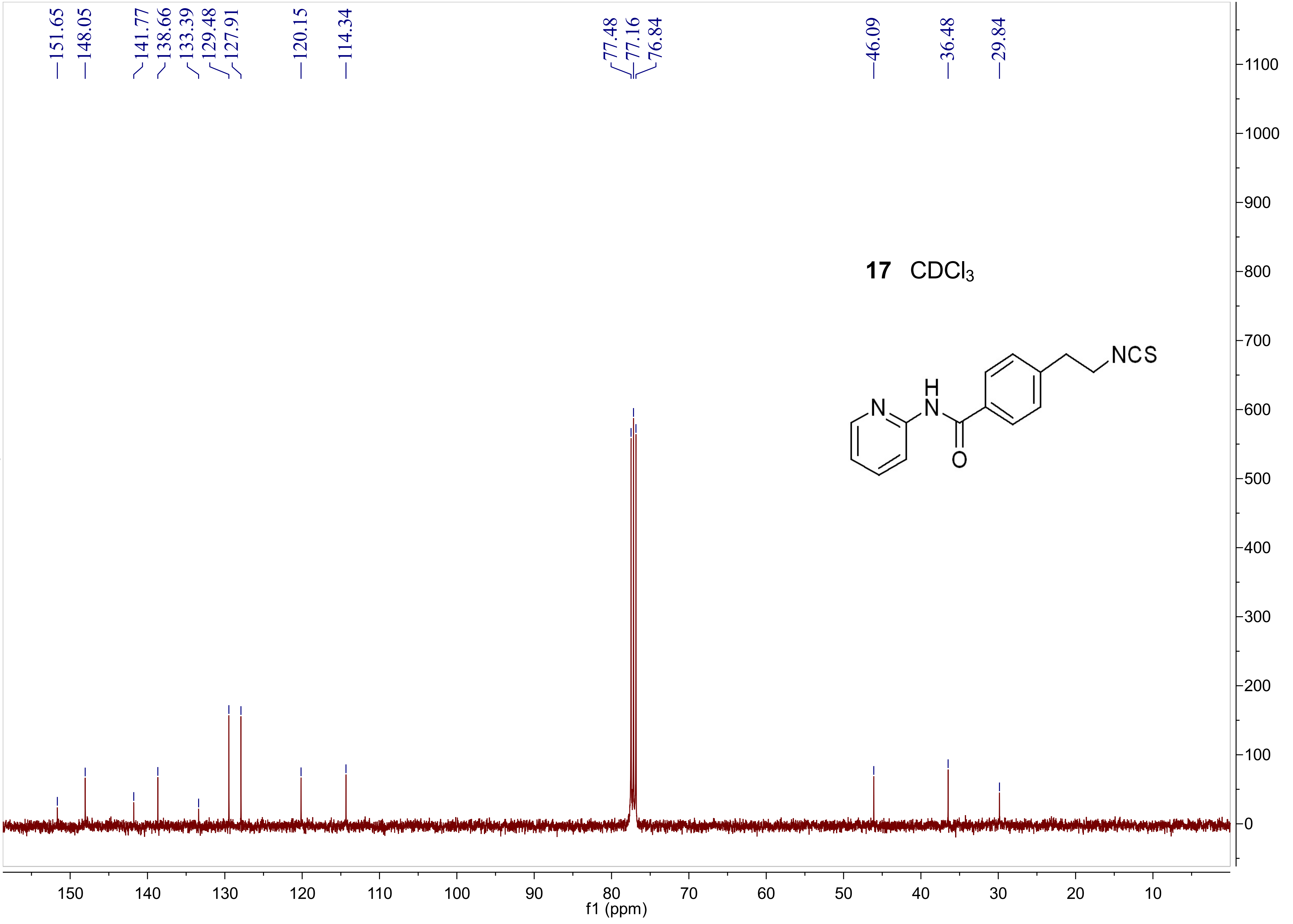
151.65
148.05
141.77
138.66
133.39
129.48
127.91
120.15
114.34
77.48
77.16
76.84
46.09
36.48
29.84
17 CDCl3
NCS
H
N
N
O
f1 (ppm)

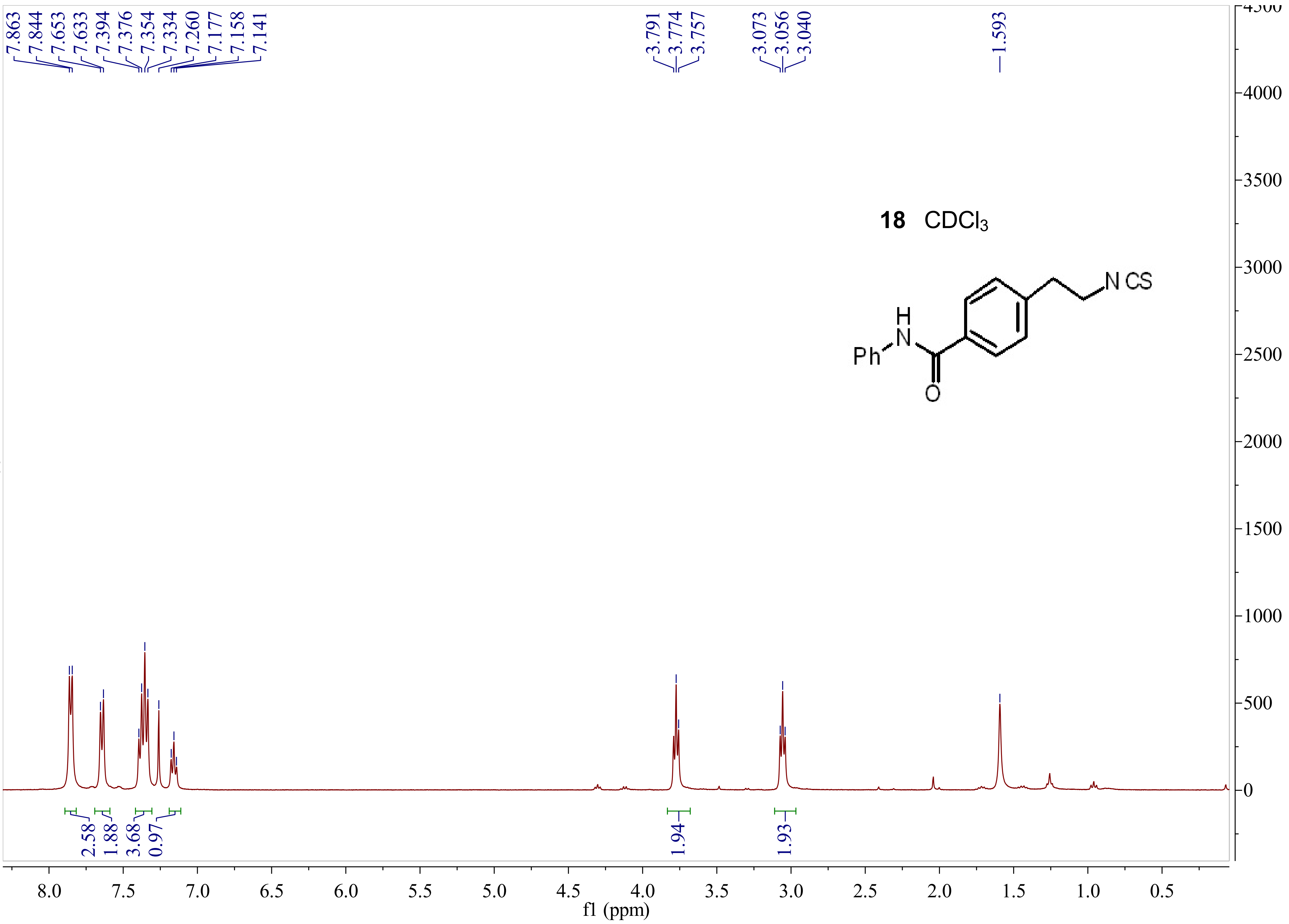
7.863
7.844
7.653
7.633
7.394
7.376
7.354
7.334
7.260
7.177
7.158
7.141
3.791
3.774
3.757
3.073
3.056
3.040
1.593
18 CDCl3
Ph
H
N
O
NCS
2.58
1.88
3.68
0.97
1.94
1.93
f1 (ppm)

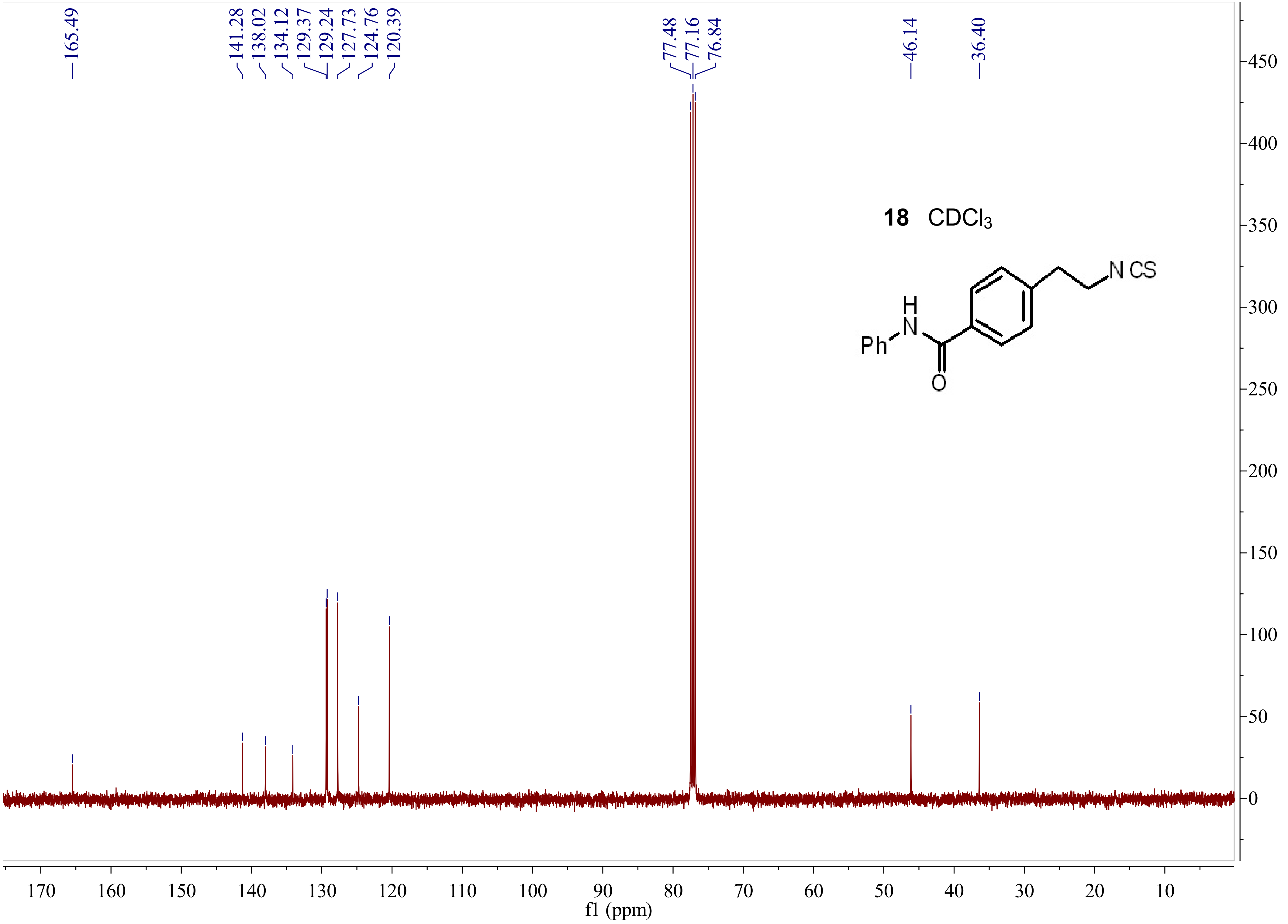

165.49
141.28
138.02
134.12
129.37
129.24
127.73
124.76
120.39
77.48
77.16
76.84
46.14
36.40
18 CDCl3
NCS
H
N
Ph
O
f1 (ppm)

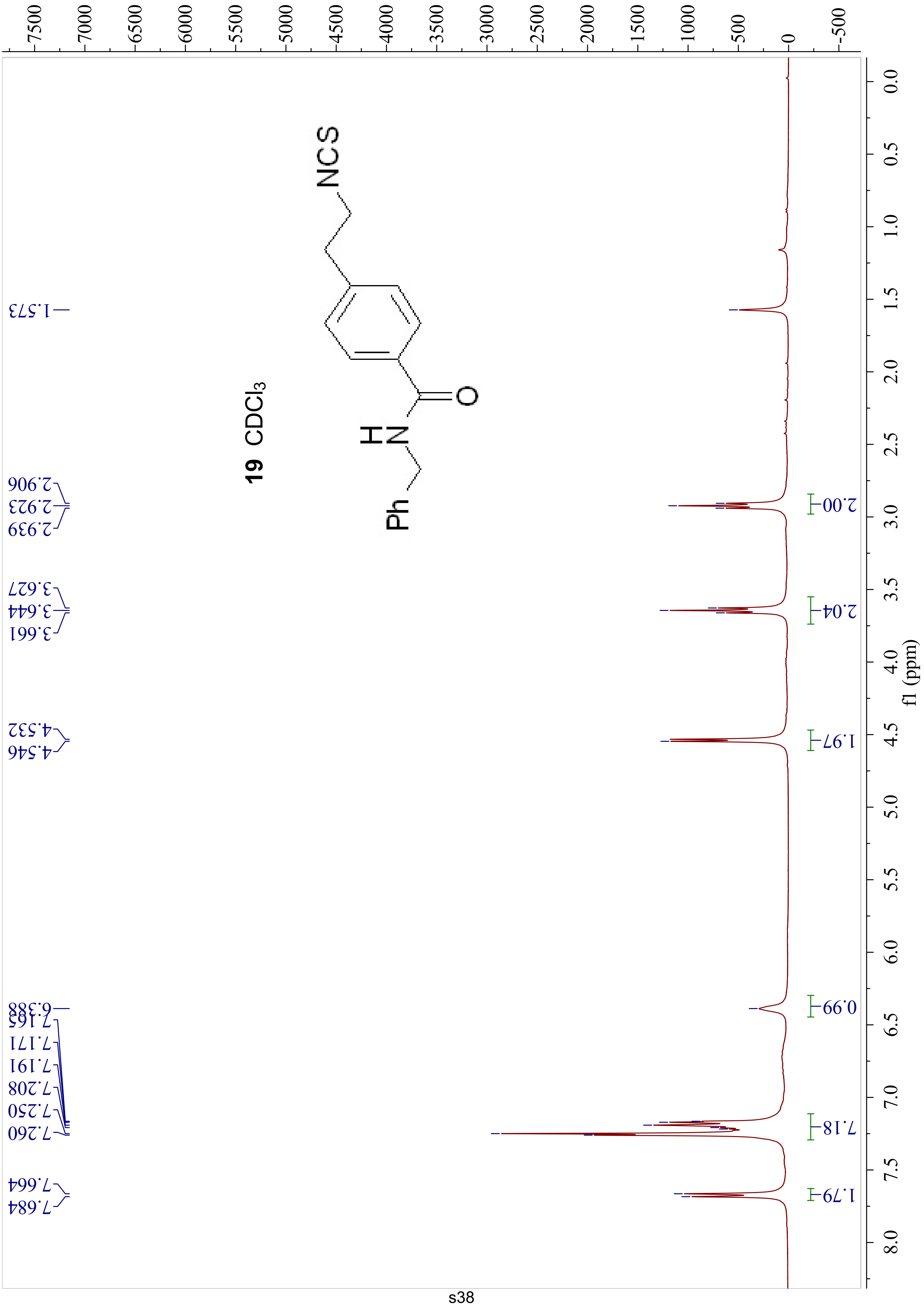

19 CDCl3
NCS
Ph
N
H
O
1.573
2.906
2.923
2.939
3.627
3.644
3.661
4.532
4.546
6.388
7.165
7.171
7.191
7.208
7.250
7.260
7.664
7.684
2.00
2.04
1.97
0.99
7.18
1.79
f1 (ppm)

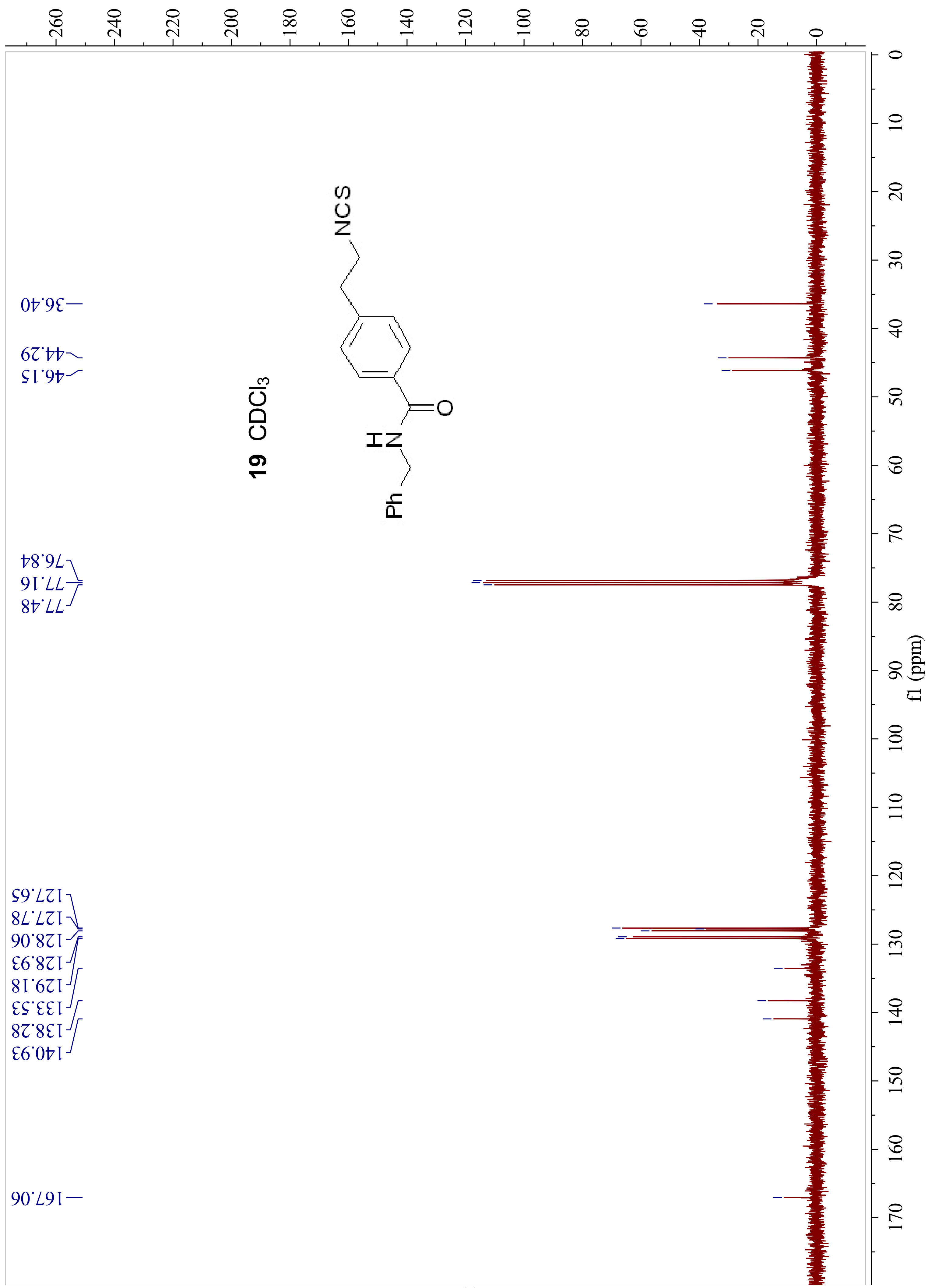
19 CDCl3
NCS
Ph
H
N
O
36.40
44.29
46.15
76.84
77.16
77.48
127.65
127.78
128.06
128.93
129.18
133.53
138.28
140.93
167.06
fl (ppm)

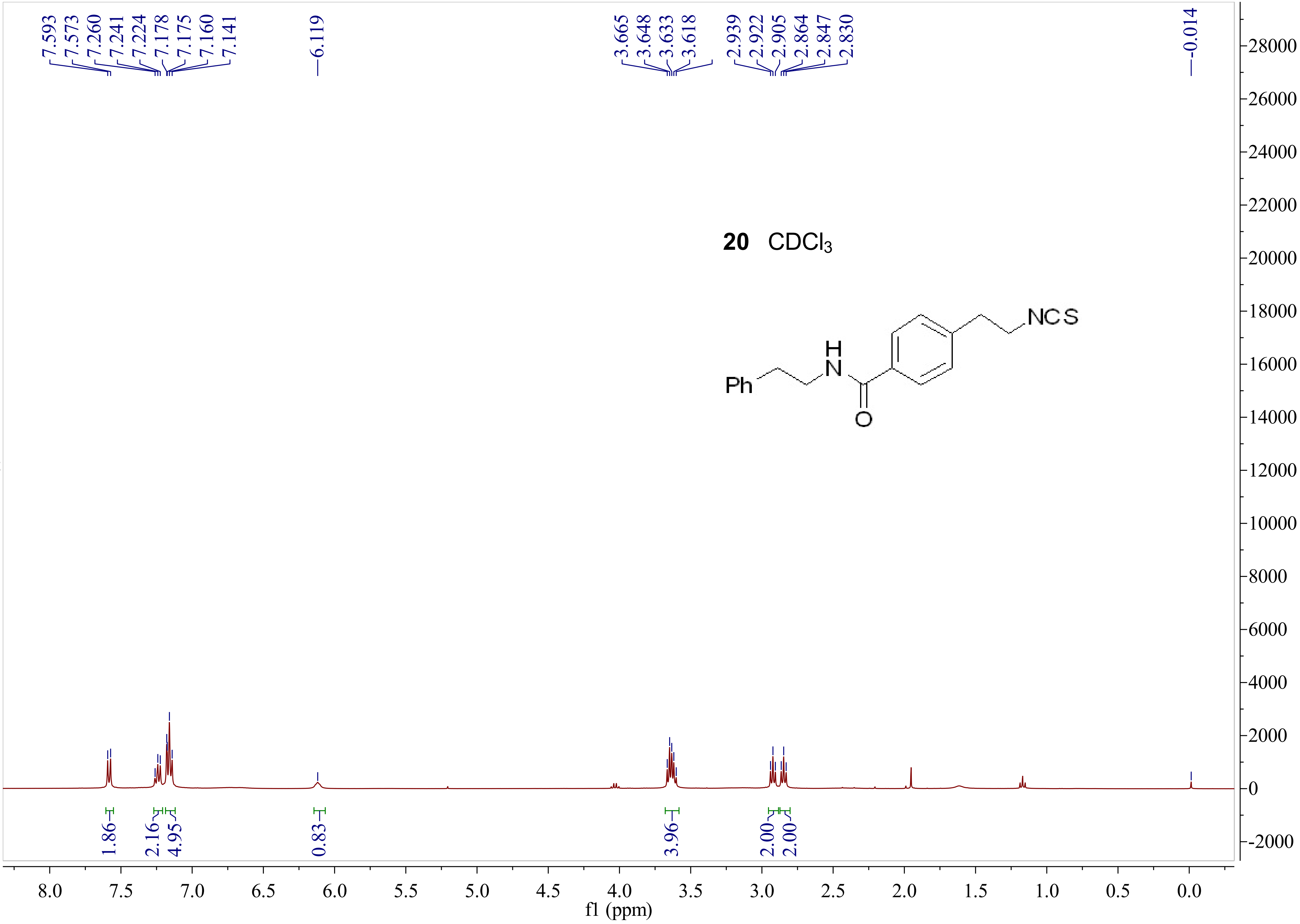
7.593
7.573
7.260
7.241
7.224
7.178
7.175
7.160
7.141
6.119
3.665
3.648
3.633
3.618
2.939
2.922
2.905
2.864
2.847
2.830
-0.014
20 CDCl3
Ph
H
N
O
NCS
1.86
2.16
4.95
0.83
3.96
2.00
2.00
f1 (ppm)

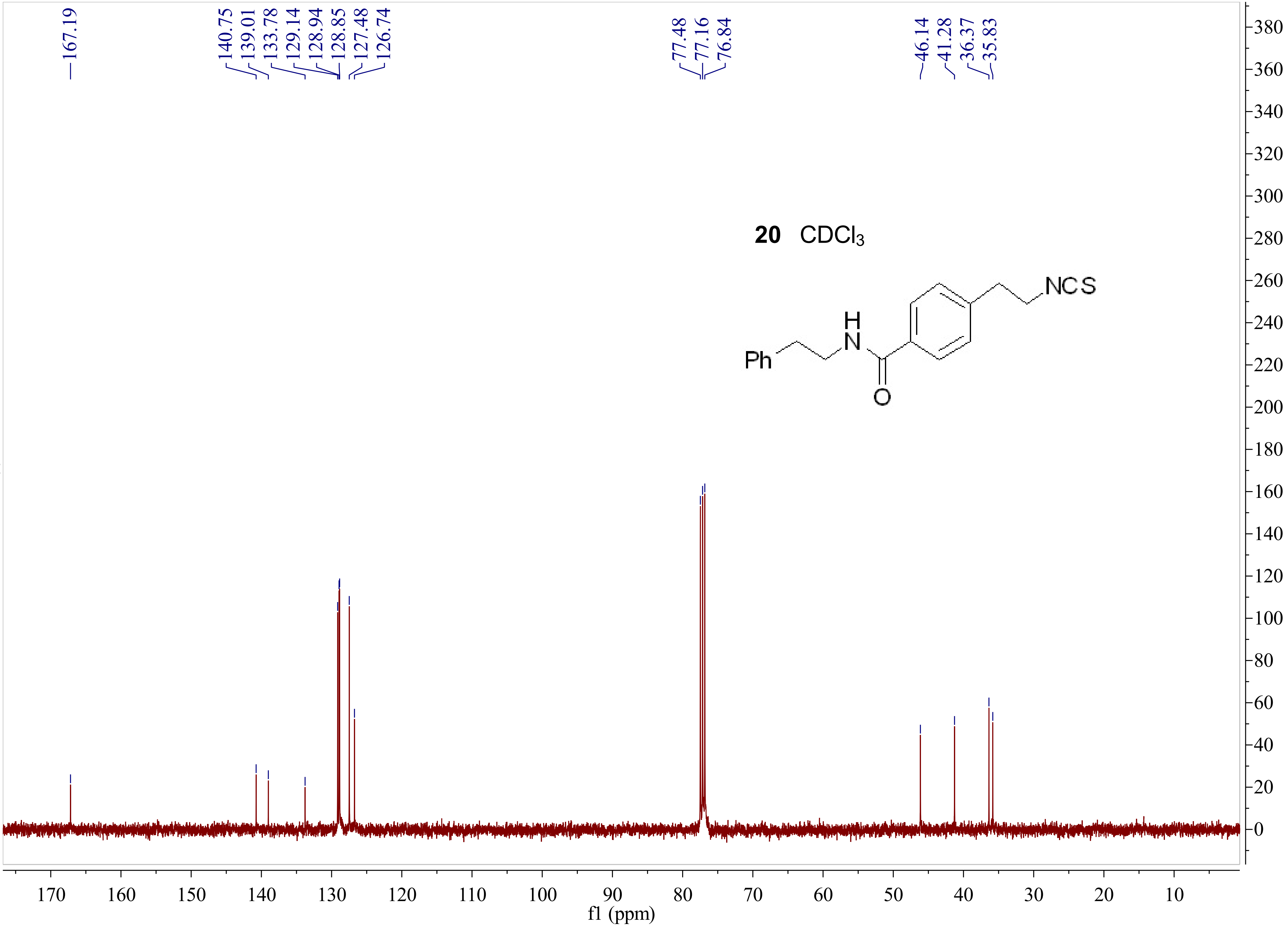
167.19
140.75
139.01
133.78
129.14
128.94
128.85
127.48
126.74
77.48
77.16
76.84
46.14
41.28
36.37
35.83
20 CDCl3
Ph
H
N
O
NCS
f1 (ppm)
